# RGB-D Image Encryption Using a Hyperchaotic System and Quaternion Gyrator Transform

**DOI:** 10.3390/e28101057

**Published:** 2026-09-25

**Authors:** Jianzhong Li, Ping Zhong, Zeying Li, Qinghua Deng, Chuying Yu, Xinan Chen, Lin Xu

**Affiliations:** 1 School of Computer and Information Engineering, Hanshan Normal University, Chaozhou 521041, China; lizj@hstc.edu.cn (J.L.); lizy@hstc.edu.cn (Z.L.); dengqh@hstc.edu.cn (Q.D.); 2 Modern Education Technology Center, Hanshan Normal University, Chaozhou 521041, China; zp@hstc.edu.cn; 3 School of Physics and Electronic Engineering, Hanshan Normal University, Chaozhou 521041, China; chyyu@hstc.edu.cn

**Keywords:** image encryption, RGB-D images, hyperchaotic systems, quaternion gyrator transform, intelligent systems

## Abstract

An RGB-D image encryption scheme is presented by combining a newly constructed 4D-ICTM hyperchaotic system with a reformulated quaternion gyrator transform (QGT). The proposed 4D-ICTM dynamics arise from the nonlinear combination of the ICMIC and Chebyshev maps with the tanh function and modulo operation. Their dynamical and statistical properties are examined using Lyapunov exponents, bifurcation diagrams, histograms, autocorrelation analysis, and correlation-dimension computation. To handle the heterogeneous RGB-D data, a dedicated preprocessing procedure converts the RGB channels and 16-bit depth data into four 8-bit components, which are represented jointly in the quaternion domain. The reformulated QGT is evaluated in terms of numerical consistency and computational cost using numerical error, SSIM, and execution-time measurements. The resulting encryption approach is further assessed through statistical analysis, adjacent-pixel correlation, floating-frequency analysis, graphic autocorrelation, and decryption-error evaluation. The experiments show resistance to the tested attack scenarios together with competitive computational performance. An encryption throughput of 43.32 Mbps is obtained at 406 cycles/byte, indicating the potential of the proposed scheme for RGB-D image encryption applications.

## 1. Introduction

With the rapid advancement of depth-sensing technologies such as Kinect, RealSense, and LiDAR, RGB-D images—comprising RGB color channels and depth maps—have become an important data modality in computer vision, robotic navigation, autonomous driving [1,2], augmented/virtual reality [3], medical diagnostics [4], and emerging metaverse applications. Compared with conventional 2D images, RGB-D data provide richer geometric structures and texture details, with depth maps typically encoded at 16-bit precision to enable accurate spatial measurements. These properties have made RGB-D data increasingly relevant to perception and decision-making tasks in intelligent systems, where the confidentiality and integrity of transmitted data are critical. For instance, in autonomous driving, RGB-D data support obstacle detection and environmental understanding, and tampering with depth information may affect system reliability or safety. In service robotics, RGB-D data can be used for 3D scene reconstruction, while leakage of indoor scanning data may compromise user privacy. Therefore, the secure and efficient transmission of heterogeneous RGB-D data remains an important challenge.

Unlike conventional color images, RGB-D data are inherently heterogeneous: RGB information consists of three correlated channels, whereas depth information is represented by a single-channel distance map. Encrypting these components separately may increase computational cost and expose individual components to potential information leakage. Moreover, intelligent applications often require timely processing of large amounts of visual data, making computational efficiency an important consideration in addition to security. These challenges motivate the development of a unified encryption framework capable of jointly protecting RGB and depth information while maintaining practical computational efficiency.

The following studies are reviewed to clarify the current research status and the remaining challenges in RGB-D image encryption. Conventional cryptographic algorithms, including AES, RSA, and ECC, are mainly intended for protecting general-purpose digital data. They can be applied to image data, but their direct application does not explicitly exploit image-specific characteristics such as spatial redundancy, local correlation, and inter-channel dependencies [5]. These characteristics have consequently encouraged the development of image-specific encryption approaches based on chaos [6,7], transform-domain operations [8,9], and hyperchaotic dynamics [10,11].

Encryption methods employing chaotic dynamics have received considerable attention owing to the sensitivity, ergodicity, pseudo-randomness, and complex dynamical behavior exhibited by chaotic systems. Initial research primarily utilized one-dimensional chaotic maps, including Logistic, Tent, and Sine maps, owing to their straightforward architecture and computational efficiency [12]. However, their relatively simple dynamics and limited chaotic parameter ranges have motivated the development of higher-dimensional chaotic systems [13,14,15,16,17,18]. Chaotic systems with three or more dimensions have subsequently been adopted for the encryption of images and multimedia data [5,19,20,21,22,23,24,25]. Hyperchaotic systems produce multiple positive Lyapunov exponents, where at least two of these indicators stay positive. This property creates greater dynamical complexity, rendering these systems appealing for image and multimedia encryption research. For example, Li employed a hyperchaotic 2D-SCM system combined with encrypted kinoforms [17]. Zhu put forward a 4D compression–encryption scheme built upon chaos theory [21]. Meng’s work reported a fractional-order five-dimensional (5D) hyperchaotic system combined with DNA coding [22]. Researchers have adopted higher-dimensional hyperchaotic systems to encrypt multi-images and color images [23,24]; meanwhile, chaotic frameworks have been extended to handle 3D and high-dimensional data including point clouds [26]. Although these methods provide effective security mechanisms, increasing system dimension and processing complexity may also introduce additional computational overhead for time-sensitive applications.

In recent years, quaternion-based transforms have been increasingly applied to the encryption of color and multiple images [27,28], because quaternion representations can jointly encode multiple channels while retaining inter-channel relationships. As one representative example, Li constructed a hybrid encryption framework that integrates the quaternion Hartley transform, two-dimensional Logistic map, and double random phase encoding within the gyrator transform (GT) domain. This method achieves better security performance yet comes with relatively heavy computational cost [27]. Ye later put forward a multi-image encryption strategy relying on the quaternion discrete fractional Hartley transform (QDFrHT), Logistic-Tent map, and Tent-Sine chaotic system [11]. However, this method only encrypts the low-frequency coefficients after discrete cosine transform (DCT), leading to noticeable distortion in the decrypted images. Shao devised a multi-image encryption approach utilizing the Logistic map alongside the quaternion gyrator transform (QGT) [10]. Another stereo-image encryption framework built upon the Logistic-Sine-coupling map and QGT was reported in [28]. In these QGT-based schemes, QGT is implemented through two quaternion Fourier transforms (Dual-QFT). Although this provides a valid computational realization, it represents the gyrator operation indirectly through frequency-domain transforms and kernel multiplication, making the geometric rotation underlying the GT less explicit. Furthermore, although the fractional order of QDFrHT and the rotation angle of QGT can theoretically enlarge the key space, these methods still face the limitation of relatively small key sizes, which poses security risks in practical scenarios.

Recently, researchers have devoted substantial interest to plaintext-associated image encryption. This strategy dynamically links encryption operations to input data, strengthening resilience against chosen-plaintext attacks and differential cryptanalysis [29,30,31,32,33,34,35,36,37]. Existing approaches may require additional plaintext-related information to be transmitted through a secure channel [31,32,35], increasing communication and key-management requirements. Similarly, optical phase-truncation-based encryption can establish plaintext dependency through phase-only masks but may require the transmission of large auxiliary data, limiting its applicability in digital environments [36]. Li proposed a plaintext-related chaotic framework that eliminates the transmission of additional plaintext information by establishing the key–plaintext relationship through chaotic pixel selection [35]. In this design, small changes in the plaintext produce significant changes in the hash value and consequently in the generated key, thereby providing strong key sensitivity and dynamic diffusion [34]. Nevertheless, such plaintext-dependent mechanisms have been mainly investigated for conventional image data, while their application to heterogeneous RGB-D data remains largely unexplored. It should also be noted that, whether auxiliary plaintext-related information or derived key information is transmitted, secure communication is required for correct decryption [31,32,35]. Therefore, integrating plaintext-dependent key generation with high-dimensional chaotic dynamics and quaternion-based processing provides a potential approach for unified protection of heterogeneous visual data.

Despite the increasing importance of RGB-D data in intelligent applications, dedicated RGB-D image encryption research remains limited. A key challenge is the inherent heterogeneity of RGB-D structures: RGB images contain three color channels, whereas depth information is represented by a single-channel distance matrix. This modality difference makes separate processing inconvenient when a unified security mechanism is desired. Although RGB-D data can be converted into 3D point clouds and protected using point-cloud encryption techniques, such conversion introduces additional computational and processing overhead. Currently, there is limited research specifically targeting RGB-D image encryption. A notable recent exception is a scheme based on finite-time synchronization of neural networks [38]. However, the separate encryption of RGB channels and depth maps increases the computational complexity of the encryption and decryption processes and may expose individual components if a single channel is compromised.

The above observations reveal three main gaps in existing research. First, existing high-dimensional chaotic encryption methods do not specifically address the unified protection of heterogeneous RGB-D data. Second, existing QGT-based encryption schemes mainly employ the Dual-QFT realization, which provides limited direct geometric interpretation of the gyrator operation. Third, plaintext-dependent key-generation mechanisms have rarely been integrated with quaternion-based processing for unified RGB-D encryption. Addressing these gaps requires a framework that jointly considers chaotic dynamics, transform-domain representation, plaintext dependency, and computational efficiency.

Motivated by these challenges, this paper proposes a holistic RGB-D image encryption scheme based on a newly constructed four-dimensional hyperchaotic system (4D-ICTM) and a reformulated quaternion gyrator transform. The framework jointly represents RGB and depth information using quaternion matrices and integrates plaintext-dependent key derivation with hyperchaotic dynamics and quaternion-domain encryption.

The primary contributions of this work are outlined below:

1. A new four-dimensional hyperchaotic system. A new four-dimensional hyperchaotic system, termed 4D-ICTM, is constructed by coupling the ICMIC map, Chebyshev map, tanh function, and modulo operation. Its dynamical complexity and statistical properties are systematically characterized using Lyapunov exponent spectra, bifurcation diagrams, phase diagrams, NIST 800-22, and additional statistical analyses.
2. A reformulated QGT with a direct computational formulation. A direct computational formulation of the QGT is derived from its definition, rather than through two quaternion Fourier transforms. The derivation preserves the geometric interpretation of gyrator rotation and provides a direct mathematical representation of the QGT. Its equivalence to the conventional QFT-based implementation is verified with SSIM = 1.0000 and a reconstruction error below 10^−14^, while the measured execution time is reduced by approximately 21.8%.
3. A unified and efficient RGB-D encryption framework. A quaternion-based framework is developed to jointly process RGB and depth information, with plaintext-dependent key derivation integrated into the 4D-ICTM and QGT-based encryption process. The proposed scheme achieves an encryption throughput of 43.3271 Mbps and an NCPB of 406 cycles/byte, and its security performance is evaluated through statistical, differential, key-sensitivity, and robustness analyses.

Overall, this work provides a unified RGB-D encryption framework that combines a newly constructed four-dimensional hyperchaotic system, a directly derived quaternion gyrator transform, and plaintext-dependent key generation. The framework is designed to jointly address the heterogeneous structure, security requirements, and computational efficiency of RGB-D data.

The rest of this paper is structured as follows. Section 2 details the construction and dynamic properties of the 4D-ICTM hyperchaotic system. Section 3 reviews the QGT and derives a direct computational formula. Section 4 presents the proposed RGB-D encryption scheme, which combines 4D-ICTM and QGT with plaintext-dependent key derivation. Experimental results and performance comparisons are provided in Section 5. Finally, Section 6 discusses limitations and future work, followed by the conclusion in Section 7.

## 2. The Construction of a New 4D Hyperchaotic Map

This section introduces the newly developed 4D-ICTM system formed by coupling the ICMIC map, Chebyshev map, tanh operation, and modulo operation, and examines its chaotic dynamics. Although the proposed 4D-ICTM utilizes existing chaotic maps as building blocks, the specific nonlinear coupling mechanism produces hyperchaotic dynamics with multiple positive Lyapunov exponents and complex trajectories.

### 2.1. Definitions of 4D-ICTM

Mathematically, the ICMIC map is defined as:

(1) $$x_{n+1}=\sin(r_{1}/x_{n}),$$

where *r*_1_ ∈ (0, ∞) [39].

The bifurcation diagram of the ICMIC map is shown in Figure 1a, together with its Lyapunov exponents in Figure 1b. These results indicate that the system complexity is strongly influenced by the value of $r_{1}$.

The Chebyshev map is mathematically defined as:

(2) $$x_{n+1}=\cos(r_{2}\cos^{-1}(x_{n})),\;\;x_{n}\in [-1,1],$$

where *r*_2_ ∈ (0, ∞) [40]. Figure 2 presents the bifurcation diagram and Lyapunov exponents of the Chebyshev map (Figure 2a,b). These results indicate that the ICMIC map possesses only a narrow chaotic region, while the Chebyshev map exhibits no chaotic behavior when $r_{2}<1$. These characteristics impose significant constraints on the applicability of both maps in cryptographic systems. Furthermore, due to having only one positive Lyapunov exponent, the two chaotic systems do not exhibit hyperchaotic states.

To enhance the randomness and expand the parameter space of chaotic systems, a hyperchaotic system, namely the 4D-ICTM, is designed and mathematically defined by Equation (3).

(3) $$\left\{\begin{array}{l} x_{n+1}=(\sin(\alpha /x_{n})+\beta z_{n})\mathrm{mod}1\\ y_{n+1}=(\cos(\lambda \cos^{-1}(y_{n}))+\gamma w_{n})\mathrm{mod}1\\ z_{n+1}=(z_{n}+\tanh(\delta x_{n}y_{n}w_{n}))\mathrm{mod}1\\ w_{n+1}=(w_{n}+y_{n}z_{n}+\tau x_{n})\mathrm{mod}1 \end{array}\right.$$

This 4D hyperchaotic system comprises four state variables and six parameters $\left(\alpha ,\beta ,\lambda ,\gamma ,\delta ,\tau \right)$. In Equation (3), $tanh\left(X\right)$ denotes the hyperbolic tangent of elements in X, while $mod\left(\cdot \right)$ denotes the modulo operation. The 4D-ICTM chaotic system integrates the strengths of the ICMIC map and the Chebyshev map, with cross-dimensional coupling fostering strong interactions among state variables, thereby increasing the nonlinearity and dynamical complexity of the system. The tanh function introduces a smooth saturation effect and contributes to the nonlinear coupling, while the mod 1 operation confines variables to the $\left[0,1\right)$ interval and induces a folding effect.

The parameters used in the subsequent analyses are set to α=3.5, β=2.8, λ=1.5, γ=4, δ=2.2, and τ=1.7. These values were selected based on subsequent parameter-sweep results, for which the system maintains three positive Lyapunov exponents over a broad parameter range. The corresponding initial conditions are $x_{1}=0.5604$, $y_{1}=0.8924$, $z_{1}=0.7845$, and $w_{1}=0.3191$.

In practical digital implementations, the continuous-valued state variables are represented using finite-precision floating-point arithmetic. Such finite precision may lead to eventual periodicity or short cycles in chaotic sequences. Therefore, the statistical properties reported below are intended to characterize the generated sequences under the specified numerical precision rather than to imply ideal randomness in infinite precision.

### 2.2. Performance Evaluation

To verify the chaotic and statistical properties of the proposed 4D-ICTM, its performance was examined using attractor phase diagrams, Lyapunov exponents, NIST statistical tests, histograms, autocorrelation, and correlation dimension analysis [41,42,43].

#### 2.2.1. Attractor Phase Diagram

The attractor phase portrait provides a visual means of analyzing the dynamic behavior of a chaotic system. A bounded and broadly distributed attractor can provide visual evidence of complex and ergodic-like dynamics; however, visual uniformity alone is not sufficient to establish cryptographic randomness.

Figure 3 presents the attractor phase diagrams of the 4D-ICTM system with the initial conditions and parameter settings given in Section 2.1. The diagrams show that the attractor points are broadly distributed throughout the bounded phase space, indicating complex dynamical behavior.

To evaluate its performance, the 4D-ICTM is compared with the four-dimensional discrete chaotic system reported in [21], whose phase diagrams are shown in Figure 4. The results demonstrate that the 4D-ICTM attractor spans a wider range of the phase space than the attractor of the system described in [21], suggesting more complex state-space dynamics.

#### 2.2.2. Lyapunov Exponents

Lyapunov exponents serve as important quantitative measures for characterizing the dynamical state of nonlinear systems. A four-dimensional nonlinear system yields four Lyapunov exponents. When at least one of these exponents is positive, the system exhibits chaotic behavior; when more than one exponent is positive, the system is classified as hyperchaotic. Compared with chaotic systems, hyperchaotic systems exhibit greater complexity and unpredictability.

Figure 5 shows the Lyapunov exponent spectrum of the 4D-ICTM system, obtained using the same initial conditions and parameter settings as those described in Section 2.2.1. Figure 6 is the corresponding bifurcation diagram.

Figure 5a–f show the Lyapunov exponent spectra of the 4D-ICTM system, obtained by varying each parameter α, β, λ, γ, δ, and τ individually while keeping the other five parameters fixed. It can be seen that across the investigated parameter ranges, LE1, LE2, and LE3 are consistently positive, while LE4 is negative, indicating that the system maintains a hyperchaotic state throughout the investigated ranges. The corresponding bifurcation diagram shows that the state points of the chaotic system are widely distributed within the interval $\left[0,1\right)$, with no obvious periodic windows or blank areas.

These results indicate that the 4D-ICTM maintains three positive Lyapunov exponents over broad parameter ranges and exhibits rich dynamical behavior. Together with the statistical analyses presented below, these results provide complementary evidence for the suitability of the generated sequences for the subsequent encryption process.

#### 2.2.3. NIST 800-22 Test

The binary sequences generated by the 4D-ICTM were subjected to the National Institute of Standards and Technology (NIST) statistical tests [41] to examine their randomness. The test suite consists of 15 subtests, with a *p*-value above 0.01 taken as the passing criterion. Table 1 reports that all binary sequences satisfy the requirements of every subtest.

**Figure 5:**
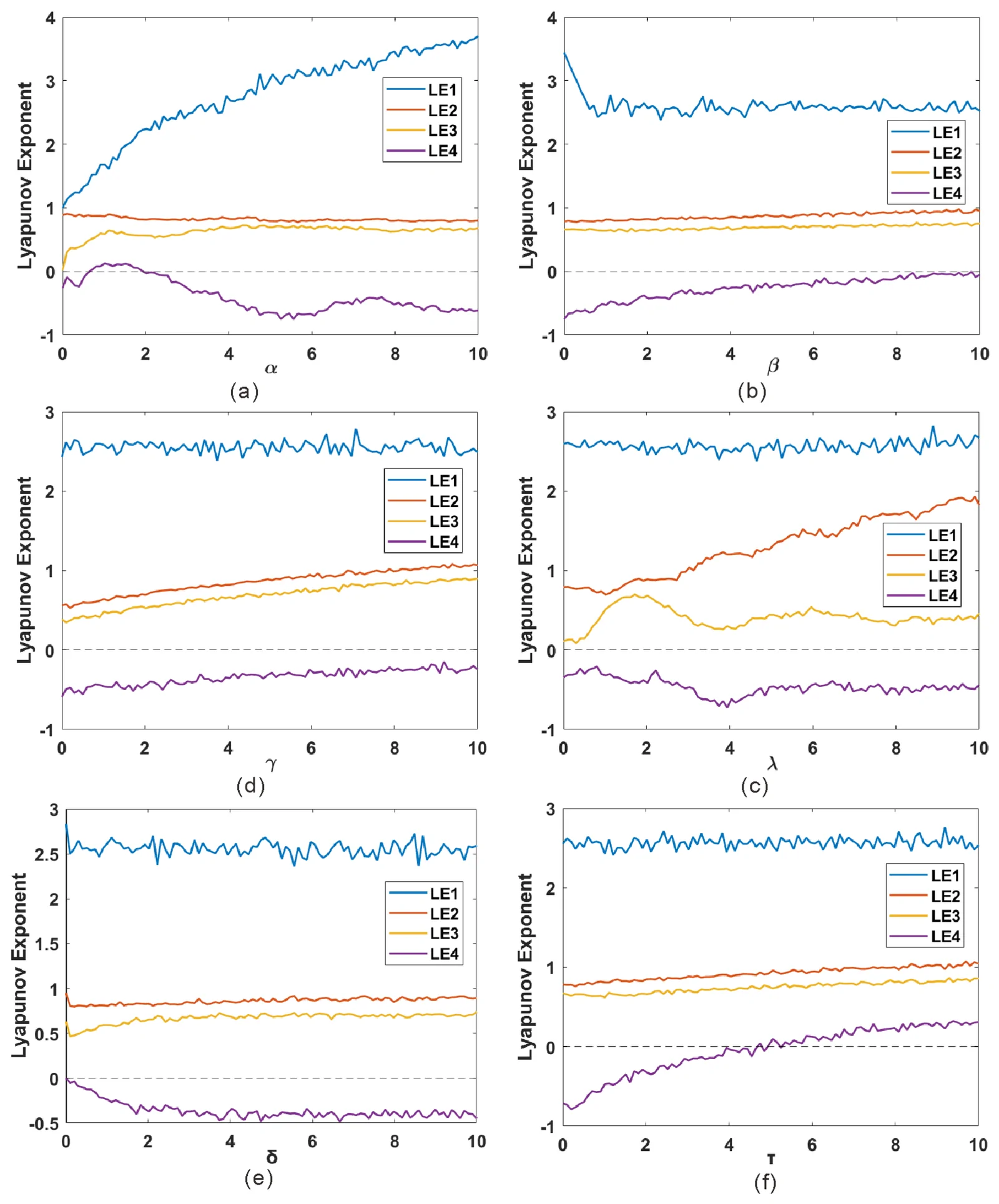
Lyapunov exponent spectra of the 4D-ICTM when varying one parameter while keeping the other five fixed. (**a**) *α* ∈ [0, 10]; (**b**) *β* ∈ [0, 10]; (**c**) *λ* ∈ [0, 10]; (**d**) *γ* ∈ [0, 10]; (**e**) *δ* ∈ [0, 10]; (**f**) *τ* ∈ [0, 10].

**Figure 6:**
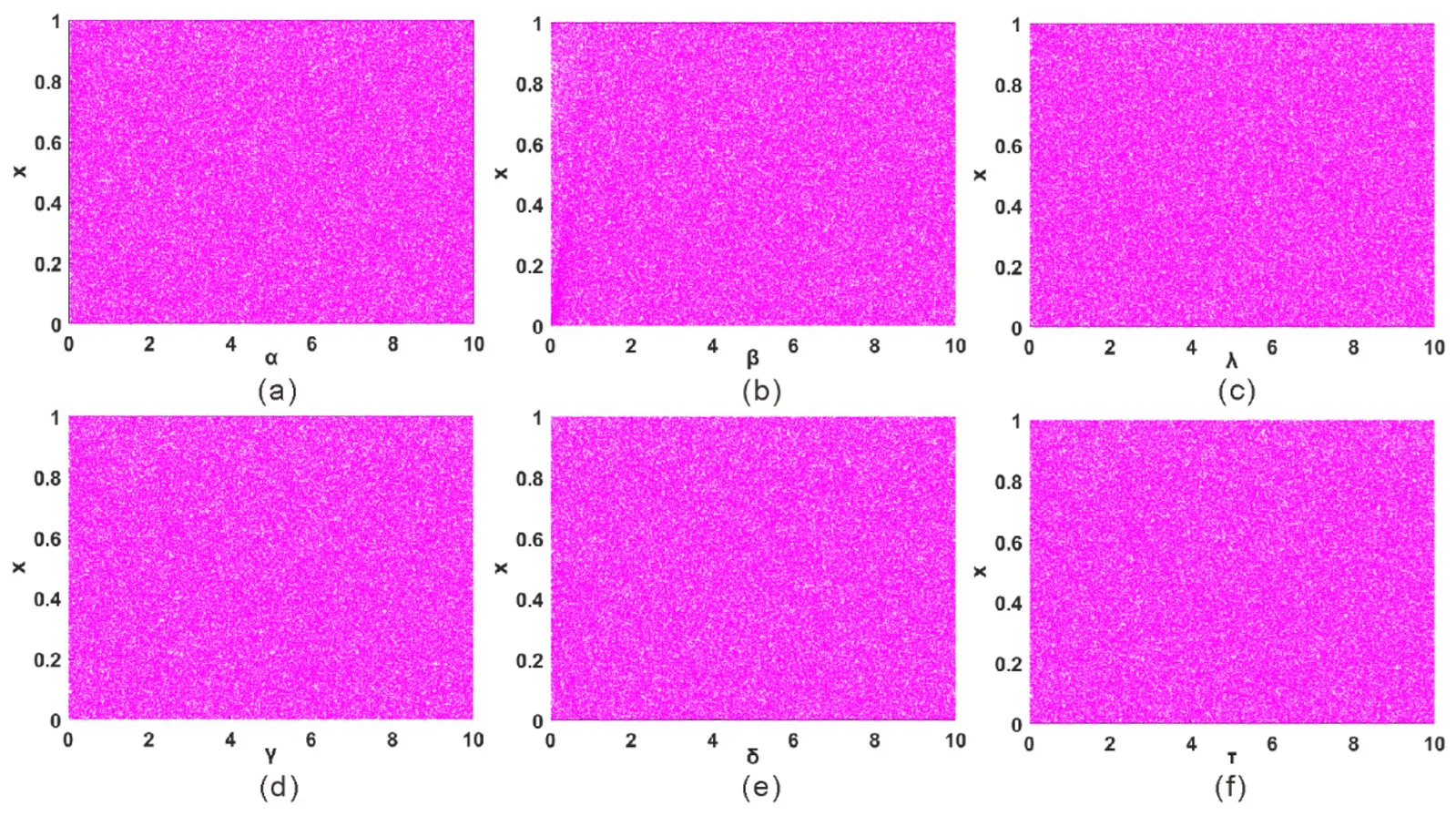
Bifurcation diagrams of the 4D-ICTM when varying one parameter while keeping the other five fixed. (**a**) *α* ∈ [0, 10]; (**b**) *β* ∈ [0, 10]; (**c**) *λ* ∈ [0, 10]; (**d**) *γ* ∈ [0, 10]; (**e**) *δ* ∈ [0, 10]; (**f**) *τ* ∈ [0, 10].

Although some p-values are relatively close to the 0.01 threshold, such as 0.017912 for the linear-complexity test of the z sequence, this value remains above the prescribed significance threshold and therefore passes the test. A relatively low p-value in a finite sample does not, by itself, indicate a weakness in the generated sequence. Instead, it reflects the statistical variation associated with finite-length testing. Therefore, the NIST results should be interpreted together with the other dynamical and statistical analyses rather than as standalone evidence of cryptographic randomness.

#### 2.2.4. Statistical Analyses

To further characterize the statistical properties of the proposed 4D-ICTM, histogram [42], autocorrelation [41], and correlation dimension analyses [43] were performed.

Figure 7 presents the probability density histograms of the four state variables. Since the modulo-1 operation confines the state variables to $\left[0,1\right)$, the ideal probability density for a uniformly distributed variable over this interval is 1. The four histograms fluctuate around this reference level, with small deviations expected from finite-sample effects, indicating that the generated state variables are approximately uniformly distributed over $\left[0,1\right)$.

Figure 8 shows the autocorrelation coefficients of the four state variables, where the lag denotes the number of sampling intervals between two sequence elements. The coefficient is 1 at zero lag and rapidly approaches zero for non-zero lags within the investigated range of 1–1000. The low non-zero-lag autocorrelation indicates weak linear dependence between samples and provides additional evidence of the low linear correlation in the generated sequences.

In addition, the correlation dimension $D_{2}$ was estimated using the Grassberger–Procaccia algorithm, and Figure 9 depicts the corresponding results. The estimated value reaches approximately $D_{2}=3.2390$ at an embedding dimension of m=6, which is below the system dimension of 4 and reflects the complex fractal structure of the attractor. This result further characterizes the complex dynamical behavior of the proposed 4D-ICTM.

The above analyses provide a broader characterization of the proposed hyperchaotic map from different statistical and dynamical perspectives, including the phase portraits, Lyapunov exponent spectra, bifurcation diagrams, NIST tests, histogram, autocorrelation, and correlation-dimension results.

## 3. Quaternion Gyrator Transform

This section first presents the fundamental theories underlying QGT and subsequently derives its computational formulation from GT.

### 3.1. Quaternion Number

Quaternions extend the concept of complex numbers and can be mathematically represented as:

(4) q=a+bi+cj+dk,

where *a*, *b*, *c*, and *d* represent real numbers [28], while *i*, *j*, and *k* denote imaginary units adhering to the following specified multiplication rules:

(5) $$i^{2}=j^{2}=k^{2}=ijk=-1,\;ij=-ji=k,\;jk=-kj=i,\;ki=-ik=j$$

When *a* = 0, *q* is termed a pure quaternion.

### 3.2. Gyrator Transform

In mathematics, the GT of a 2D function *g*(*x*, *y*) with respect to rotation angle *θ* can be expressed as:

(6) $$G(u,v)=GT^{\theta }(g(x,y))=\frac{1}{\left|\sin\theta \right|}\int \int g(x,y)\exp\left[j2\pi \frac{\left(uv+xy)\cos\theta -(uy+vx\right)}{\sin\theta }\right]dxdy,$$

where *GT^θ^*() denotes the gyrator transform [44]. Since *F*(*u*,*v*) is a complex function, it can also be expressed as:

(7) GTθ(g(x,y))=Re(GTθ(g(x,y)))+jIm(GTθ(g(x,y)))

Let *T* = exp(*j*2*π*((*uv* + *xy*)cos*θ* − (*uy* + *vx*))/sin*θ*). Using Euler’s formula exp(*jx*) = cos*x* + *j*sin*x*, Equation (6) can be rewritten as:

(8) $$\begin{array}{l} GT^{\theta }(g(x,y))=\frac{1}{\left|\sin\theta \right|}\int \int g(x,y)(\cos T+j\sin T)dxdy\\ \;\;\;\;\;=\frac{1}{\left|\sin\theta \right|}\int \int g(x,y)\cos Tdxdy+\frac{j}{\left|\sin\theta \right|}\int \int g(x,y)\sin Tdxdy \end{array}$$

By comparing Equations (7) and (8), the real and imaginary components of the gyrator spectrum *G*(*u*,*v*) can be written as follows:

(9) Re(GTθ(g(x,y)))=1|sinθ|∫∫g(x,y)cosTdxdy Im(GTθ(g(x,y)))=1|sinθ|∫∫g(x,y)sinTdxdy

The IGT is equivalent to the GT evaluated at −θ and is given by:

(10) $$g(x,y)=IGT^{\theta }(G(u,v))=GT^{-\theta }(G(u,v)),$$

where IGT() is the inverse gyrator operator.

### 3.3. Definition of Quaternion Gyrator Transform

The non-commutativity of quaternion multiplication leads to multiple formulations of the QGT. For a two-dimensional quaternion-valued function (*g_Q_*(*x*, *y*)), its right-sided QGT at an angle (*θ*) is defined as follows:

(11) $$G_{Q}(u,v)=QGT^{\theta }(g_{Q}(x,y))=\frac{1}{\left|\sin\theta \right|}\int \int g_{Q}(x,y)\exp\left[\mu 2\pi \frac{\left(uv+xy)\cos\theta -(uy+vx\right)}{\sin\theta }\right]dxdy$$

where ***μ*** is a pure unit quaternion [28]. In [10,28], two left-sided quaternion Fourier transforms (QFT) were used to calculate QGT.

The inverse transform of the right-side QGT, denoted as IQGT, is defined as:

(12) $$g_{Q}(x,y)=IQGT^{\theta }(G_{Q}(u,v))=QGT^{-\theta }(G_{Q}(u,v))$$

Assuming that the height and width of *g_Q_*(*x*, *y*) are *M* and *N*, respectively, since the computational cost of a 2D QFT is O(4*MN*log_2_*MN*), the total cost of the method for computing QGT in [10,28] is O(8*MN*log_2_*MN*). However, this method decomposes the QGT into two frequency-domain operations and a pointwise multiplication with a kernel function, which obscures its rotational essence and increases the difficulty of theoretical understanding. Therefore, the following derivation presents a method for computing the QGT using the Gyrator transform.

### 3.4. Calculation for the QGT of a Quaternion Function

Applying Euler’s Formula for quaternions, exp(***μ****x*) = cos*x* +***μ***sin*x* [44], Equation (11) can be reformulated as:

(13) $$G_{Q}(u,v)=QGT^{\theta }(g_{Q}(x,y))=\frac{1}{\left|\sin\theta \right|}\int \int g_{Q}(x,y)(\cos T+\mu \sin T)dxdy$$

Let the general unit pure quaternion be ***μ*** = *ρi* + *ξj* + *ηk* (*ρ*, *ξ* and *η* are real numbers), and let *g_Q_*(*x*, *y*) = *g*_0_(*x*, *y*) + *g*_1_(*x*, *y*)*i* + *g*_2_(*x*, *y*)*j* + *g*_3_(*x*, *y*)*k*. For brevity, in the following symbols *g_Q_*, *g*_0_, *g*_1_, *g*_2_ and *g*_3_ will be used to represent *g_Q_*(*x*, *y*), *g*_0_(*x*, *y*), *g*_1_, *g*_2_ and *g*_3_(*x*, *y*), respectively. Substituting ***μ*** and *g_Q_*(*x*, *y*) into Equation (13) yields:

(14) $$\begin{array}{l} G_{Q}(u,v)=\frac{1}{\left|\sin\theta \right|}\int \int g_{Q}\cos Tdxdy+\frac{1}{\left|\sin\theta \right|}\int \int g_{Q}\mu \sin Tdxdy\\ \;\;\;=\frac{1}{\left|\sin\theta \right|}\int \int \left(g_{0}+g_{1}i+g_{2}j+g_{3}k\right)\cos Tdxdy+\frac{1}{\left|\sin\theta \right|}\int \int \left(g_{0}+g_{1}i+g_{2}j+g_{3}k\right)(\rho i+\xi j+\eta k)\sin Tdxdy \end{array}$$

By applying the quaternion properties given in Equation (5) to Equation (14), Equation (15) can be derived as follows:

(15) $$G_{Q}(u,v)=G_{Q0}(u,v)+iG_{Q1}(u,v)+jG_{Q2}(u,v)+kG_{Q3}(u,v),$$

where the following applies:

(16) GQ0(u,v)=Re[GTθ(g0)]−ρIm[GTθ(g1)]−ξIm[GTθ(g2)]−ηIm[GTθ(g3)]GQ1(u,v)=Re[GTθ(g1)]+ρIm[GTθ(g0)]+ηIm[GTθ(g2)]−ξIm[GTθ(g3)]GQ2(u,v)=Re[GTθ(g2)]+ξIm[GTθ(g0)]−ηIm[GTθ(g1)]+ρIm[GTθ(g3)]GQ3(u,v)=Re[GTθ(g3)]+ηIm[GTθ(g0)]+ξIm[GTθ(g1)]−ρIm[GTθ(g2)]

From Equations (15) and (16), it can be seen that the QGT spectrum of *g_Q_* can be obtained by performing the GT operation on *g*_0_, *g*_1_, *g*_2,_ and *g*_3_. The reconstructed function *g′_Q_*(*x*, *y*) is obtained through the application of IQGT to the expression in Equation (15). Notably, the IQGT itself can also be rigorously derived from the IGT as follows:

(17) $$\begin{array}{l} g_{Q}^{\prime }(x,y)=IQGT(G_{Q}(u,v))=QGT^{-\theta }(G_{Q}(u,v))\\ \;\;\;=QGT^{-\theta }[G_{Q0}(u,v)+iG_{Q1}(u,v)+jG_{Q2}(u,v)+kG_{Q3}(u,v)]\\ \;\;\;=g_{0}^{\prime }(x,y)+g_{1}^{\prime }(x,y)i+g_{2}^{\prime }(x,y)j+g_{3}^{\prime }(x,y)k, \end{array}$$

where the following applies:

(18) g0′(x,y)=Re(Ig0(x,y))−ρIm(Ig1(x,y))−ξIm(Ig2(x,y))−ηIm(Ig3(x,y))g1′(x,y)=Re(Ig1(x,y))+ρIm(Ig0(x,y))+ηIm(Ig2(x,y))−ξIm(Ig3(x,y))g2′(x,y)=Re(Ig2(x,y))+ξIm(Ig0(x,y))−ηIm(Ig1(x,y))+ρIm(Ig3(x,y))g3′(x,y)=Re(Ig3(x,y))+ηIm(Ig0(x,y))+ξIm(Ig1(x,y))−ρIm(Ig2(x,y))

where the following applies:

(19) $$Ig_{0}(x,y)=IGT^{\theta }(G_{Q0}),Ig_{1}(x,y)=IGT^{\theta }(G_{Q1}),Ig_{2}(x,y)=IGT^{\theta }(G_{Q2}),Ig_{3}(x,y)=IGT^{\theta }(G_{Q3})$$

In Equation (19), the terms *G_Q_*_0_, *G_Q_*_1_, *G_Q_*_2_ and *G_Q_*_3_ correspond to the *G_Q_*_0_(*u*,*v*), *G_Q_*_1_(*u*,*v*), *G_Q_*_2_(*u*,*v*) and *G_Q_*_3_(*u*,*v*), respectively. As demonstrated in the preceding derivation steps, the QGT and IQGT of a quaternion matrix can be efficiently computed using conventional GT and IGT algorithms.

In the derived formulations, the QGT is computed through four GT operations, and the IQGT through four IGT operations. Since a single GT or IGT has a computational cost of O(2*MN*log_2_*MN*) [45], the total cost of the QGT is O(8*MN*log_2_*MN*), and that of the IQGT is also O(8*MN*log_2_*MN*), which is consistent with the method in [10,28]. However, in our approach, the GT is applied directly to compute the QGT and the IGT to obtain the IQGT, thereby circumventing the indirect procedure in [10,28] that derives these transforms through dual QFTs. This direct strategy preserves the clarity of interpretation and streamlines the mathematical framework, presenting the QGT in a cohesive formal structure that maintains internal consistency.

We perform numerical experiments on four standard 512 × 512 grayscale test images, namely Bridge, Boat, Cameraman, and Couple, to quantitatively evaluate the practical performance of the proposed method against the conventional Dual-QFT implementation. These images were assigned to the real (*q*_0_) and three imaginary (*q*_1_, *q*_2_, *q*_3_) components of the quaternion signal, respectively. Table 2 summarizes the performance comparison in terms of numerical equivalence, measured by Maximum Absolute Error (MAE) [46] and Structural Similarity Index Measure (SSIM) [28], as well as computational efficiency, evaluated by Execution Time. An SSIM value lies in the range of [0, 1], and a higher value implies better visual quality.

As shown in Table 2, both methods achieve reconstruction errors in the order of 10^−15^ and SSIM values of 1.0000, which confirms that the proposed direct calculation is numerically equivalent to the Dual-QFT method within the limits of floating-point precision. Although the proposed method exhibits a marginally higher rounding error, it achieves numerically accurate reconstruction under the tested conditions. Furthermore, the proposed method reduces the measured execution time by approximately 21.8%, from 0.0551 s for the Dual-QFT implementation to 0.0431 s for the proposed direct calculation. This efficiency gain is attributed to the streamlined algebraic structure, which reduces the overhead associated with the dual-transform decomposition.

## 4. The Proposed Holistic RGB-D Image Encryption and Decryption Scheme Based on 4D-ICTM and QGT

Figure 10 presents the block diagrams of the proposed RGB-D image encryption and decryption processes. In the encryption method, the RGB-D image containing two different types of data is first preprocessed to produce four images of the same data type. The resulting preprocessed images are then disturbed using the 4D-ICTM. Subsequently, the disturbed images are represented as a holistic vector in quaternion form and transformed by the QGT. Finally, the 4D-ICTM is applied to the transformed image to produce the Ciphertext image. The decryption process reverses these operations to recover the original RGB-D image.

### 4.1. RGB-D Image Preprocessing and Restoration Algorithm

To achieve holistic encryption of an RGB-D image, a preprocessing algorithm is first used to convert the depth map and RGB image, which have different data formats, into four images of the identical data format required for encryption. The preprocessing reorganizes the original RGB channels and the two 8-bit components of the 16-bit depth map into four 8-bit images, without discarding any RGB-D information. During decryption, a restoration algorithm is then applied to recover the original depth map and RGB image from the ciphertext. Thus, the preprocessing and restoration operations form a reversible data-reorganization process. This section describes the two symmetric processes in detail.

#### 4.1.1. The Preprocessing Algorithm

Let *rgb* and *dm* denote the 24-bit RGB image and 16-bit depth map components of the RGB-D image, respectively, both with dimensions of height *M* and width *N*. The preprocessing procedure is described as follows:

1. First, the *rgb* image is decomposed into red, green, and blue channels, denoted as *rc*, *gc*, and *bc*, respectively, all of which are 8-bit images. Then, the depth map *dm* is split into high 8-bit image *dh* and low 8-bit image *dl* using Equation (20):
  (20) dh=floor(dm/256),      dl=dmmod256
  where floor() denotes the floor function, which performs downward rounding to the nearest integer. The two preceding operations yield five 8-bit images with a uniform data format: *rc*, *gc*, *bc* from RGB channel decomposition, and *dh*, *dl* from depth map bit-splitting.
2. To enable quaternion representation of all image data, the five 8-bit images obtained from Step 1 need to be merged into four 8-bit images. Specifically, *dl* is divided into four equal-sized sub-images along the rows, and each of these four sub-images is then integrated row-wise into one of the other four images. This row-wise partition only redistributes the low 8-bit depth data among the four output images and does not change their pixel values. Since *M* may not be a multiple of 4, the number of zero-padding rows *Z* is first determined through a modulo operation to ensure that the total number of rows *M*′ of the zero-padded *dl* is a multiple of 4:
  (21) Z=(4−mod(M,4))mod4
  As indicated by Equation (21), *Z* can be 0, 1, 2, or 3.
3. A zero-padded matrix *dl*′ is constructed with row count *M*′ = *M* + *Z*, following the formula:
  (22) $$dl\prime =\left[\begin{array}{l} dl\\ 0_{Z\times N} \end{array}\right]$$
  Here, 0*_Z_*_×*N*_ denotes a zero matrix of *Z* rows and *N* columns, *dl*′ is a matrix of size *M*′ × *N*, and *M*′ is a multiple of 4. The added rows contain only zeros and are removed during the restoration process; therefore, zero padding does not alter the original depth data.
4. The matrix *dl*′ is row-wise partitioned into four sub-matrices *s*1, *s*2, *s*3, and *s*4, each with dimensions (*M*′/4) × *N*.
5. By merging *rc* with *s*1, *gc* with *s*2, *bc* with *s*3, and *dh* with *s*4 along the rows, four output images *g*_0_, *g*_1_, *g*_2_ and *g*_3_, are obtained:
  (23) $$g_{0}=\left[\begin{array}{l} rc\\ s1 \end{array}\right],g_{1}=\left[\begin{array}{l} gc\\ s2 \end{array}\right],g_{2}=\left[\begin{array}{l} bc\\ s3 \end{array}\right],g_{3}=\left[\begin{array}{l} dh\\ s4 \end{array}\right]$$
  Here, the sizes of *g*_0_, *g*_1_, *g*_2_, and *g*_3_ are all *R* × *N*, where *R* = *M* + *M*′/4.

Figure 11 illustrates a preprocessing instance for the RGB-D image NYU0009 of size 427 × 561. According to the algorithm, in the preprocessing stage, the number of zero-padded rows is 1, resulting in 8-bit merged images *g*_0_, *g*_1_, *g*_2,_ and *g*_3,_ each with a size of 534 × 561.

#### 4.1.2. The Restoration Algorithm

This algorithm will reversibly recover the color image *rgb* and depth map *dm* from the merged images *g*_0_, *g*_1_, *g*_2,_ and *g*_3_. In the image reconstruction process, the number of zero-padded rows *Z* and the original image row count *M* must first be calculated from the number of rows *R* in the merged image before proceeding with image recovery. The detailed steps are as follows:

1. From the preprocessing algorithm, it is known that *Z* is the unique integer satisfying 0 ≤ *Z* ≤ 3 and the following conditions:

(24) $$(4R-Z)\mathrm{mod}5=0\;\mathrm{and}\;Z=(4-(\frac{4R-Z}{5})\mathrm{mod}4)\mathrm{mod}4$$

Thus, the steps for computing *Z* and *M* are described in Algorithm 1.
**Algorithm 1:** Compute *Z* and *M*Input: *g*_0_  [*R*, ~] = size(*g*_0_)  for *Z* from 0 to 3 do    // Determine whether *Z* is a valid zero-padding row count    if ((4×*R* − *Z*) mod 5 == 0) && (*Z*==(4 − ((4 ×*R* − *Z*) / 5) mod 4) mod 4) then      *M* = *R* − *Z* // Calculate the number of rows *M* of the original image      break    end if  end forOutput: *Z* and *M*

2. According to the following formulas, extract sub-blocks s1, s2, s3 and s4 of size (R-M) × N from g0, g1, g2 and g3, respectively:

(25) $$s1=g_{0}(M+1:R,:),\;s2=g_{1}(M+1:R,:),\;s3=g_{2}(M+1:R,:),\;s4=g_{3}(M+1:R,:)$$

3. Stack the sub-blocks *s*1, *s*2, *s*3 and *s*4 along the rows to reconstruct the zero-padded *dl*′ of size 4(*R* − *M*) × *N*: *dl*′ = [*s*1; *s*2; *s*3; *s*4]. Here, the symbol ‘;’ denotes a row-wise (vertical) stacking operation.
4. Apply Equation (26) to recover the images *rc*, *gc*, *bc*, *dh*, and *dl*:

(26) $$\begin{array}{l} rc=g_{0}(1:M,:),\;gc=g_{1}(1:M,:),\;bc=g_{2}(1:M,:)\\ dh=g_{3}(1:M,:),\;dl=dl\prime (1:M,:) \end{array}$$

5. Reconstruct the color image *rgb* by recombining *rc*, *gc* and *bc*, and calculate the depth map *dm* using Equation (27):

(27) dm=dh×256+dl

### 4.2. The RGB-D Image Encryption Scheme

Consistent with Section 4.1.1, let *rgb* and *dm* represent the color component and depth map (both sized *M* × *N*) of an RGB-D image, respectively. The overall design of the proposed RGB-D image encryption scheme is outlined below:

1. Based on the preprocessing method described in Section 4.1.1, the heterogeneous RGB-D data are converted into a uniform format, producing four images—*g*_0_, *g*_1_, *g*_2_, and *g*_3_—with consistent data format and size of *R* × *N*.
2. The mean pixel values of plaintext images *g*_0_, *g*_1_, *g*_2_, and *g*_3_ are first computed. Each mean value is divided by a random integer within [1, 512], and a small constant *t* is introduced to avoid division by zero. Modulo operations implemented on these processed values produce the initial values *x*1, *y*1, *z*1, and *w*1 of the 4D-ICTM chaotic system, based on which an *R* × *N* random chaotic integer matrix *dMta* is generated for encryption. The random integers utilized in this step are internal and discarded after computing *x*1, *y*1, *z*1, and *w*1. These values do not constitute any part of the encryption key and are never transmitted. These random integers are then discarded immediately upon completion of the above operations.
3. XOR operations between the chaotic matrix *dMta* and each of the images *g*_0_, *g*_1_, *g*_2,_ and *g*_3_ are performed, followed by normalization of the resulting images.
4. Using Equation (16), the quaternion vector, composed of the four disturbed and normalized images obtained in Step 3, is transformed by QGT to obtain four spectral coefficient matrices *G_Q_*_0_, *G_Q_*_1_, *G_Q_*_2_, and *G_Q_*_3_.
5. The IGT is applied to the spectrum matrices using Equation (19) to generate the intermediate images *Ig*_0_, *Ig*_1_, *Ig*_2_, and *Ig*_3_.
6. The mean amplitude of the intermediate images is calculated and used as the initial value to input into the 4D-ICTM chaotic system to generate the chaotic sequence *pStr*, followed by scrambling operations on the intermediate images using *pStr*.
7. The scrambled intermediate images are reconstructed using Equation (18) to obtain *rg*_0_, *rg*_1_, *rg*_2,_ and *rg*_3_, followed by merging the four reconstructed images into a matrix of size 2*R* × 2*N*.
8. The merged matrix is quantized into an 8-bit image to produce the ciphertext image *ERGBD*.

The pseudocode of the proposed encryption approach is presented in Algorithm 2.
**Algorithm 2:** RGB-D image encryption algorithmInput: *rgb, dm, α*1, *β*1, *λ*1, *γ*1, *δ*1, *τ*1, *θ*, *α*2, *β*2, *λ*2, *γ*2, *δ*2, *τ*2 //Step 1: Preprocess the RGB-D data according to Section 4.1.1 [*g*_0_, *g*_1_, *g*_2_, *g*_3_] = preprocessing (*rgb*, *dm*) //Step 2: Generate the initial values of the first 4D-ICTM system [*x*1, *y*1, *z*1, *w*1] = mod(mean(*g*_0_, *g*_1_, *g*_2_, *g*_3_)/randi([1, 512]), 1) + *t* //Step 3: Generate four chaotic sequences [*X*1, *Y*1, *Z*1, *W*1] = 4DICTM(*x*1, *y*1, *z*1, *w*1, *α*1*, β*1*, λ*1*, γ*1*, δ*1*, τ*1, *R* × *N*) //Step 4: Generate the chaotic integer matrix *dMta* = reshape(mod(floor((*X*1 + *Y*1 + *Z*1 + *W*1) × 10^6^), 256), *R*, *N*) //Step 5: Perform XOR diffusion *g*_0_ = normalize(bitxor(*g*_0_, *dMta*)),    *g*_1_ = normalize(bitxor(*g*_1_, *dMta*)), *g*_2_ = normalize(bitxor(*g*_2_, *dMta*)),    *g*_3_ = normalize(bitxor(*g*_3_, *dMta*)) //Step 6: Construct the quaternion vector and perform QGT *g_Q_* = *g*_0_ + *g*_1_*i* + *g*_2_*j* + *g*_3_*k*,     [*G_Q_*_0_, *G_Q_*_1_, *G_Q_*_2_,*G_Q_*_3_] = QGT(*g_Q_*, *θ*) //Step 7: Perform IGT on the four QGT components *Ig*_0_ = IGT(*G_Q_*_0_, *θ*),  *Ig*_1_ = IGT(*G_Q_*_1_, *θ*),  *Ig*_2_ = IGT(*G_Q_*_2_, *θ*),  *Ig*_3_ = IGT(*G_Q_*_3_, *θ*) //Step 8: Generate the initial values of the second 4D-ICTM system [*x*2, *y*2, *z*2, *w*2] = mean (*Ig*_0_, *Ig*_1_, *Ig*_2_, *Ig*_3_) //Step 9: Generate four chaotic sequences [*X*2, *Y*2, *Z*2, *W*2] = 4DICTM(*x*2, *y*2, *z*2, *w*2, *α*2, *β*2, *λ*2, *γ*2, *δ*2, *τ*2, *R* × *N*) //Step 10: Generate the permutation sequence *pStr* = (*X*2 + *Y*2 + *Z*2 + *W*2)/4,        [~, *pos*] = sort(*pStr*) //Step 11: Convert the four components into 1D matrices *Ig*_0__1D = reshape(*Ig*_0_, 1, *R* × *N*),       *g*_1__1D = reshape(*Ig*_1_, 1, *R* × *N*), *Ig*_2__1D = reshape(*Ig*_2_, 1, *R* × *N*),       *g*_3__1D = reshape(*Ig*_3_, 1, *R* × *N*) //Step 12: Scramble the four components using *pos* *pIg*_0_ = *Ig*_0__1D(*pos*),    *pIg*_1_ = *Ig*_1__1D(*pos*),    *pIg*_2_ = *Ig*_2__1D(*pos*),    *pIg*_3_ = *Ig*_3__1D(*pos*) //Step 13: Reshape the scrambled components *Ig*_0_ = reshape(*pIg*_0_, *R*, *N*),   *Ig*_1_ = reshape(*pIg*_1_, *R*, *N*),   *Ig*_2_ = reshape(*pIg*_2_, *R*, *N*),   *Ig*_3_ = reshape(*pIg*_3_, *R*, *N*) //Step 14: Reconstruct the four components according to Equation (18) *rg*_0_ = real(*Ig*_0_) − *ρ* × imag(*Ig*_1_) − *ξ* × imag(*Ig*_2_) − *η* × imag(*Ig*_3_),    *rg*_1_ = real(*Ig*_1_) + *ρ* × imag(*Ig*_0_) + *η* × imag(*Ig*_2_) − *ξ* × imag(*Ig*_3_) *rg*_2_ = real(*Ig*_2_) + *ξ* × imag(*Ig*_0_) − *η* × imag(*Ig*_1_) + *ρ* × imag(*Ig*_3_),   *rg*_3_ = real(*Ig*_3_) + *η* × imag(*Ig*_0_) + *ξ* × imag(*Ig*_1_) − *ρ* × imag(*Ig*_2_) //Step 15: Merge and quantize the four components *ERGBD* = quantize([*rg*_0_ *rg*_1_; *rg*_2_ *rg*_3_], 0, 255)Output: the encrypted image *ERGBD*

The parameters of the proposed RGB-D image encryption scheme, including *x*1, *y*1, *z*1, *w*1, *α*1, *β*1, *λ*1, *γ*1, *δ*1, *τ*1, *θ*, *x*2, *y*2, *z*2, *w*2, *α*2, *β*2, *λ*2, *γ*2, *δ*2, and *τ*2, constitute a broad key space. This characteristic contributes to the overall security of the cryptosystem. To ensure secure key separation and reproducibility, the proposed RGB-D image encryption system employs a two-level key hierarchy. This design also establishes a plaintext-dependent encryption mechanism without requiring the transmission of any additional plaintext-related information to the receiver. The keys are organized into two distinct levels, denoted as KEY1 and KEY2, each serving a specific function in the encryption process.

- KEY1 = {*x*1, *y*1, *z*1, *w*1, *x*2, *y*2, *z*2, *w*2} is derived during the encryption process from the plaintext and intermediate spectrum features and is treated as session-specific secret information.
- KEY2 = {α1, β1, λ1, γ1, δ1, τ1, θ, α2, β2, λ2, γ2, δ2, τ2} represents the system control parameters, which are fixed and securely configured at both the sender and receiver in advance.

For every encryption session, the generated KEY1 values are delivered to the receiving party over a confidential, authenticated channel. This channel is typically established using asymmetric key exchange mechanisms or within a TLS-protected tunnel. Using these session-specific keys together with the pre-shared KEY2, the receiver can accurately regenerate the chaotic sequences required for decryption without access to the original plaintext. The random integers adopted to produce *x*1, *y*1, *z*1 and *w*1 are internal and discarded after use. They form no component of KEY1 or KEY2 and are never transmitted to the receiver.

It should be noted that the focus of this work lies in the algorithmic design and security evaluation of the encryption process, while the establishment of secure communication channels for key delivery follows standard cryptographic practice and is beyond the scope of this study.

### 4.3. Decryption Process

The developed cryptosystem adopts a symmetric encryption scheme. During decryption, all operations performed during encryption are reversed, and the detailed workflow is presented below.

1. First, normalize the encrypted image *ERGBD*, and then calculate its height 2*R* and width 2*N*.
2. Let *rg*_0_ = *ERGBD* [1:*R*, 1:*N*], *rg*_1_ = *ERGBD* [1:*R*, *N* + 1:2*N*], *rg*_2_ = *ERGBD* [*R* + 1:2*R*, 1:*N*] and *rg*_3_ = *ERGBD* [*R* + 1:2*R*, *N* + 1:2*N*].
3. Construct a quaternion vector using *rg*_0_, *rg*_1_, *rg*_2_, and *rg*_3_ and perform QGT on it with a rotation angle of *θ* to obtain *eG_Q_*_0_, *eG_Q_*_1_, *eG_Q_*_2,_ and *eG_Q_*_3_.
4. Using the decryption keys *x*2, *y*2, *z*2, *w*2, *α*2, *β*2, *λ*2, *γ*2, *δ*2 and *τ*2, we determine the permutation index pos according to steps (9)–(10) of the encryption process.
5. By applying the IGT with a rotation angle *θ* according to Equation (19), the transformed images *sIg*_0_, *sIg*_1_, *sIg*_2_ and *sIg*_3_ are obtained from *eG_Q_*_0_, *eG_Q_*_1_, *eG_Q_*_2_ and *eG_Q_*_3_. Subsequently, each of these images is mapped into a 1D sequence, yielding *sIg*_0__1D, *sIg*_1__1D, *sIg*_2_ _1D and *sIg*_3__1D.
6. Subsequently, permute *sIg*_0__1D, *sIg*_1__1D, *sIg*_2__1D and *sIg*_3__1D back to their original position using the following equations:

(28) $$\begin{array}{l} dIg_{0}\_1D(pos)=sIg_{0}\_1D,\;\;\;\;\;\;dIg1\_1D(pos)=sIg_{1}\_1D,\\ dIg_{2}\_1D(pos)=sIg_{2}\_1D,\;\;\;\;\;\;dIg_{3}\_1D(pos)=sIg_{3}\_1D \end{array}$$

7. Map *dIg*_0__1D, *dIg*_1__1D, *dIg*_2__1D and *dIg*_3__1D into 2D matrices of size *R* × *N*, respectively, to obtain *dIg*_0_, *dIg*_1_, *dIg*_2_ and *dIg*_3_.
8. By applying Equation (18) to *dIg*_0_, *dIg*_1_, *dIg*_2_ and *dIg*_3_, the results *drg*_0_, *drg*_1_, *drg*_2_ and *drg*_3_ are obtained.
9. First, following steps (3)–(4) of the encryption process, the random chaotic integer matrix *dMta* is generated using the decryption keys *x*1, *y*1, *z*1, *w*1, *α*1, *β*1, *λ*1, *γ*1, *δ*1 and *τ*1. Subsequently, *drg*_0_, *drg*_1_, *drg*_2_ and *drg*_3_ are quantized into 8-bit images, and the quantized results are XORed with *dMta* to obtain *dg*_0_, *dg*_1_, *dg*_2_ and *dg*_3_ using Equation (29).

(29) $$\begin{array}{l} dg_{0}=quantize(drg_{0},0,255)⊕dMta,\;\;\;\;\;\;dg_{1}=quantize(drg_{1},0,255)⊕dMta,\\ dg_{2}=quantize(drg_{2},0,255)⊕dMta,\;\;\;\;\;\;dg_{3}=quantize(drg_{3},0,255)⊕dMta, \end{array}$$

where ⊕ denotes the XOR operator.

10. Finally, by applying the algorithm described in Section 4.1.2 to *dg*_0_, *dg*_1_, *dg*_2,_ and *dg*_3_, the color component *rgb* and the depth image *dm* of the decrypted RGB-D image are recovered.

## 5. Experimental Results

A series of tests was conducted to verify the performance of our designed encryption scheme. The hardware platform was a Dell laptop (Dell Inc., Round Rock, TX, USA) equipped with an Intel^®^ Core™ i9-14900HX processor at 2.20 GHz, 32 GB RAM, running Windows 11 (25H2) and MATLAB R2024a (MathWorks, Natick, MA, USA). As shown in Figure 12, two RGB-D images of size 427 × 561 from the SUNRGBD dataset (“NYU0001” and “NYU0300”) and one RGB-D image of size 480 × 640 from the Scenes dataset (“scene_09/00666”) were used for experimental testing [47,48,49,50,51,52,53]. In Figure 12a–c show the color image, depth map, and 3D point cloud visualization (azimuth 10°, elevation −80°) of NYU0001, respectively; Figure 12d–f show the corresponding results for NYU0300; and) Figure 12g–i show those for 00666. The parameters are configured as follows: *α*1 = 3.5*, β*1 = 2.7, *λ*1 = 1.5, *γ*1 = 4.5, *δ*1 = 3.2, *τ*1 = 2.3, *θ* = 11π/12, *α*2 = 5, *β*2 = 3, λ2 = 1.8, γ2 = 2, δ2 = 1.2,τ2 = 3.5 and ***μ*** = (*i* + *j* + *k*)/3^1/2^. The constant *t* is set to 10^−15^. The SSIM was employed to measure the similarity between the decrypted RGB-D image and the original one. Since *x*1, *y*1, *z*1, *w*1, *x*2, *y*2, *z*2, and *w*2 are computed during the encryption process, their initial values differ when encrypting different RGB-D images. For example, the values of *x*1, *y*1, *z*1, *w*1, *x*2, *y*2, *z*2 and *w*2 calculated from “NYU0001” were 0.485477809862555, 0.483036421522485, 0.450837574977384, 0.383220774807417, 0.690322546356771, 0.460655233226517, 0.429734462290743 and 0.440484433422396, respectively.

Figure 13 illustrates the encrypted and decrypted RGB-D results obtained using the proposed method with the correct keys. In Figure 13a, e, and i are the ciphertext images of “NYU0001”, “NYU0300”, and “00666”, respectively. These encrypted images appear as irregular noise, from which no meaningful information can be discerned. Figure 13b–d show the decrypted color image, decrypted depth map, and 3D point cloud visualization of “NYU0001”. Figure 13f–h present the corresponding decrypted results for “NYU0300”. And Figure 13j–l display the corresponding decrypted results for “00666”. The decrypted images match the original RGB-D images, with SSIM values of 1 for all components, including both color images and depth maps.

### 5.1. Key Space and Sensitivity Analysis

Cryptographic keys underpin the security of image encryption cryptosystems. Researchers generally use the key-space size to assess resistance to brute-force attacks, with 2^100^ widely regarded as the minimum recommended threshold [19]. In the proposed scheme, the secret key consists of 21 parameters: *x*1, *y*1, *z*1, *w*1, *α*1, *β*1, *λ*1, *γ*1, *δ*1, *τ*1, *θ*, *x*2, *y*2, *z*2, *w*2, *α*2, *β*2, *λ*2, *γ*2, *δ*2 and *τ*2.

The key space is determined by the precision of these parameters. Each of the 20 parameters (excluding *θ*) is represented in 64-bit double precision and maintains 15 significant digits after the decimal point, resulting in a search space of 10^15^ for each parameter. Thus, their combined contribution is (10^15^)^20^ = 10^300^. For *θ*, with a sensitivity of 10^3^, the search space is 10^3^. Consequently, the overall key space of the proposed system reaches 10^303^, far exceeding the benchmark of 2^100^, which indicates a high theoretical search complexity under the assumed parameter precision. This value represents the theoretical upper bound of the key space. Since the theoretical key-space calculation is based on the numerical precision of the parameters, the effective key space in a finite-precision implementation may be smaller than the theoretical upper bound. Nevertheless, the large key space makes an exhaustive search impractical from a computational perspective.

Key sensitivity is another essential property of a secure encryption algorithm. A secure cryptosystem should exhibit high key sensitivity, such that slight changes in the secret key lead to completely different encryption and decryption outcomes. To evaluate this property, each parameter was perturbed slightly (e.g., *x*1 = *x*1 + 10^−15^, *y*1 = *y*1 + 10^−15^, …) while keeping the remaining keys unchanged. Instead of presenting decrypted visual diagrams for every single parameter perturbation, several representative visual examples shown in Figure 14, Figure 15 and Figure 16 are provided, and quantitative SSIM metrics are summarized in Table 3. As observed, even such small deviations yield completely unrecognizable images, which also fail to reconstruct valid 3D visualizations. Moreover, the SSIM values reported in Table 3 are close to zero, confirming the high key sensitivity of the proposed scheme.

### 5.2. Histogram Analysis

The histogram of an image reflects the distribution of its pixel intensities. For plaintext images, pixel values usually cluster within certain gray-level ranges, leading to uneven histograms. To resist statistical attacks, however, a secure encryption algorithm should generate encrypted images whose histograms are uniformly distributed. Figure 17 demonstrates that the histograms of the cipher images exhibit an approximately flat intensity distribution, in marked contrast to the highly concentrated distributions of the original RGB-D images. This uniformity significantly hinders statistical analysis by potential attackers.

The uniformity of pixel distribution in cipher images can be examined through histogram visualization for qualitative assessment and the Chi-square (χ^2^) test for quantitative verification, whose statistical formulation is given in Equation (30) [54].

(30) $$\chi^{2}=\sum_{i=0}^{L-1}\frac{{\left(O_{i}-E_{i}\right)}^{2}}{E_{i}}$$

In Equation (30), *L* specifies the number of intensity levels, with *L* = 256 for an 8-bit grayscale image. *O_i_* is the measured frequency associated with intensity level *i*, whereas *E*_i_ is the frequency predicted by the theoretical distribution.

The Chi-square distribution table is referenced for the statistical assessment. With 255 degrees of freedom, the 5% critical value is 293 [54]. The χ^2^ statistics reported in Table 4 are obtained from the three ciphertext images in Figure 13. For the proposed scheme, none of the test results provide sufficient evidence to reject the null hypothesis. At the 5% significance level, all encrypted images show *p*-values above 0.05, confirming their pixel distribution uniformity. This characteristic indicates that the ciphertext exhibits a relatively uniform gray-level distribution under the tested conditions.

To further inspect the gray-level distribution in the cipher data, we carried out floating frequency analysis [55] on the cipher output of “NYU0001”. Figure 18 plots the row-wise and column-wise floating frequencies computed with 256 × 256 sliding windows. The mean row and column floating frequencies are 162.08 and 162.06, respectively, which are close to the theoretical expectation of 162.01 distinct gray levels for a 256-pixel window. The results indicate stable floating-frequency behavior of the ciphertext.

### 5.3. Correlation of Adjacent Pixels

Images differ from text data in that neighboring pixels generally exhibit strong spatial correlations along the horizontal, vertical, and diagonal directions. If encrypted images retain such correlations, an attacker may exploit a small portion of ciphertext data to infer the remaining information and eventually recover the plaintext, making the system vulnerable to statistical attacks. To counter this threat, a robust image encryption scheme should suppress pixel correlations in all three directions. In this work, the correlation between adjacent pixels of plaintext and ciphertext images is quantitatively evaluated. Specifically, we randomly selected 5000 pixel pairs along the horizontal, vertical, and diagonal directions, and their correlation coefficients ($r_{xy}$) were computed according to the following formula [17]:

(31) $$r_{xy}=\frac{\mathrm{cov}(x,y)}{\sqrt{D(x)}\sqrt{D(y)}}$$

Here, cov(*x*,*y*) = (1/*N*)Σ(*x_i_* − *E*(*x*))(*y_i_* − *E*(*y*)), *E*(*x*) = (1/*N*)Σ*x_i_*, and *D*(*x*) = Σ(*x_i_* − *E*(*x*))^2^. The variables *x* and *y* represent two neighboring pixels indexed by *i*. Larger absolute values of *r_xy_* correspond to stronger pixel correlation. The calculated correlation values are compiled in Table 5, and the associated distributions of the RGB-D sample “NYU0001” are plotted in Figure 19. As observed in Table 5 and Figure 19, the original RGB channels and depth map exhibit coefficients near 1, whereas the corresponding cipher data are concentrated around zero, showing that neighboring-pixel dependence is largely eliminated after encryption. Figure 19 reveals a clear linear relationship between neighboring pixels in the original RGB-D image, whereas the cipher image presents a flat distribution with negligible correlation.

Graphic autocorrelation analysis [55] was also performed to evaluate the residual spatial correlation in the plaintext and ciphertext. Figure 20 shows the results for the R channel, depth map, and ciphertext image of “NYU0001”. The plaintext R channel and depth map exhibit pronounced central peaks and relatively high background levels, indicating strong spatial correlation. This is particularly evident in the depth map, which contains large smooth regions. In contrast, the ciphertext shows a sharp central peak with a much lower background level, indicating that the spatial correlation is substantially reduced after encryption. The corresponding peak-to-background ratios are 4.10, 5.95, and 1.37 × 10^3^ for the R channel, depth map, and ciphertext, respectively. The much higher ratio of the ciphertext indicates that its off-center autocorrelation is negligible relative to the central peak, providing further evidence of the effective suppression of spatial correlation.

### 5.4. Information Entropy Analysis

Information entropy (IE) measures the degree of uncertainty in the distribution of image data. It can be computed using Equation (32) [54].

(32) $$H(f)=\sum_{i=1}^{255}P(f_{i})\log_{2}\frac{1}{P(f_{i})},$$

Here, $H\left(X\right)$ quantifies the information entropy, and L specifies the number of possible pixel values. The values of L are 256 for 8-bit images and 65,536 for 16-bit images. In addition, $p\left(x_{i}\right)$ gives the probability of occurrence of pixel value $x_{i}$.

According to Equation (32), entropy reaches its theoretical maximum when all pixel values occur with equal probability. Specifically, the maximum entropy is 8 for an 8-bit image and 16 for a 16-bit image. Therefore, entropy values approaching these limits indicate a more uniform pixel-value distribution. Table 6 reports the entropy measurements for the original RGB-D image and its encrypted counterpart. Compared with the relatively low entropy values of the four plaintext channels, the corresponding cipher channels exhibit values close to the theoretical limit of 8. These findings indicate that the proposed encryption method makes the cipher pixel distribution more uniform and increases its statistical unpredictability, thereby improving resistance to entropy-based cryptanalysis. It is worth noting that although the depth image is 16-bit, its entropy remains relatively low due to pixel values being concentrated within a limited range.

### 5.5. Differential Attack Analysis

Analogous to key sensitivity evaluation, differential attack testing examines the sensitivity of the cryptosystem to variations in the plaintext. In this scenario, an adversary is presumed to gain temporary access to the encryption system. Two widely adopted metrics for such assessments include the number of pixel change rate (NPCR) and unified average change intensity (UACI), with their corresponding calculations given below [56]:

(33) $$\begin{array}{l} NPCR=\frac{\sum_{ij}D(i,j)}{M\times N}\times 100\%\\ UACI=\frac{1}{M\times N}[\sum_{i,j}\frac{C_{1}(i,j)-C_{2}(i,j)}{255}]\times 100\% \end{array}$$

Here, *M* and *N* stand for the row count and column count of the tested image, respectively. *C*_1_ and *C*_2_ correspond to the cipher images produced before and after a one-pixel alteration in the plaintext, respectively. For a perfect cryptosystem, the theoretical NPCR is roughly 99.6094%, whereas the expected UACI is approximately 33.4635% [56]. These two metrics serve as essential benchmarks for assessing the encryption scheme’s sensitivity to differential changes. A higher NPCR signifies greater sensitivity to plaintext changes, while a UACI value close to the expected range indicates a substantial change in the ciphertext intensity distribution.

In the experiments, the RGB-D plaintext images were processed by keeping one of their two constituent components (color/depth) unaltered while randomly modifying a single pixel in the other component. Specifically, either the color component remained unchanged with a single pixel alteration in the depth map, or vice versa. Table 7 presents the performance evaluation metrics of the proposed encryption scheme. It can be observed from Table 7 that the obtained NPCR and UACI values closely match their theoretical values. These results indicate high sensitivity of the ciphertext to the tested single-pixel changes.

### 5.6. Plaintext Attacks Analysis

Cryptanalytic threats mainly include ciphertext-only, known-plaintext, chosen-ciphertext, and chosen-plaintext attacks. Among these attack modes, the chosen-plaintext attack provides a particularly demanding test of the proposed image cryptosystem. Resistance to chosen-plaintext attacks is therefore an important criterion for evaluating the security of image encryption schemes. To evaluate resistance to chosen-plaintext attacks, pure-color images (e.g., all-white or all-black) are commonly used as test inputs. The results, shown in Figure 21 and Table 8, indicate that the encrypted images exhibit almost no correlation with their originals, and their histograms are approximately an ideal random distribution. For the tested all-white and all-black plaintexts, the resulting ciphertexts exhibit near-zero adjacent-pixel correlations and entropy values close to 8, indicating that the obvious statistical characteristics of the tested plaintexts are not retained in the ciphertexts. Even when plaintexts contain salient structures, the corresponding ciphertexts do not exhibit obvious visual or adjacent-pixel statistical characteristics of the tested plaintexts. With the tested metrics approaching their theoretical reference values, the results indicate that the proposed scheme reduces the retention of obvious plaintext-related statistical characteristics under the tested chosen-plaintext scenarios.

### 5.7. Noise and Data Loss Attacks Analysis

During image data transmission, noise interference and data loss are potential risks that must be considered. Taking the ciphertext of “NYU0001” as an illustrative example, we assessed the robustness of our proposed cryptosystem against noise corruption and data truncation. In the noise-resilience test, the ciphertext image was subjected to 20% and 30% salt-and-pepper noise, as illustrated in Figure 22a,e. The corresponding decryption results are shown in Figure 22: (b) and (c) present the decrypted RGB image and depth map from (a), with SSIM values of 0.1303 and 0.0680, respectively, while (d) shows the reconstructed 3D point cloud. Similarly, (f) and (g) display the decrypted RGB image and depth map from (e), with SSIM values of 0.0874 and 0.0407, respectively, and (h) presents the corresponding 3D visualization.

In the data loss test, the ciphertext image was subjected to occlusion attacks covering 1/4 and 1/3 of its area, as shown in Figure 23a,e. The decrypted results are given in Figure 23: (b) and (c) correspond to the decrypted RGB image and depth map from (a), with SSIM values of 0.1100 and 0.0402, while (d) illustrates the reconstructed point cloud. Similarly, (f) and (g) correspond to the decrypted results of (e), with SSIM values of 0.0788 and 0.0360, and (h) presents the corresponding 3D reconstruction.

To further measure the discrepancy between reconstructed images and the original counterparts under these attack scenarios, the decryption error was calculated using the MAE. The resulting errors for the 20% and 30% salt-and-pepper noise attacks were 12.72% and 16.14% for the RGB component, and 9.90% and 14.05% for the depth map, respectively. For the 1/4 and 1/3 data loss attacks, the corresponding errors were 14.44% and 17.30% for the RGB component, and 11.78% and 15.31% for the depth map, respectively. These results are consistent with the visual observations in Figure 22 and Figure 23, where the main structural features remain recognizable despite the degradation caused by noise and data loss.

Notably, although the ciphertext images suffer from information degradation under noise and occlusion attacks, the decrypted images preserve the main structural features of the plaintext, and their reconstructed 3D representations remain recognizable.

### 5.8. Efficiency Analysis

In cryptographic design, speed performance is another critical metric. The computational complexity of the proposed encryption method can be divided into four main components. The first stage is the preprocessing of heterogeneous RGB-D images. Since the number of zero-padding rows is at most three, the primary cost arises from splitting and recombining the depth map, which involves one floor() operation, one mod() operation, and four concatenations of sub-images of size approximately *MN*/4. The resulting complexity is O(3*MN*). The second stage is image confusion, which includes four mean calculations, one chaotic sequence generation, and one floor, mod, and XOR operation each. As shown in Equation (3), the chaotic sequence generation requires O(4*MN*), yielding a total complexity of O(11*MN*). The third stage processes four images in vectorized form, involving four normalization operations and one QGT operation, with a complexity of O(8*RN*log_2_*RN* + 4*RN*). The fourth stage is image permutation, comprising four mean operations, one chaotic sequence generation, one quicksort, four sequence permutations, one inverse QGT, and four quantization operations, resulting in O(4*RN* + 4*RN* + *RN*log_2_*RN* + 4*RN* + 8 *RN*log_2_*RN* + 4*RN*) = O(9*RN*log_2_*RN* + 16*RN*). Therefore, the total computational cost of the encryption process is O(3*MN* + 11*MN* + 17*RN*log_2_*RN* + 20*RN*). Since *R* ≈ 5/4*MN*, the overall complexity can be approximated as O(39*MN* + 105/4*MN*log_2_(5/4*MN*)).

By applying a similar analysis, the computational cost of the decryption algorithm is obtained as O(50*MN* + 105/4*MN*log_2_(5/4*MN*)), which indicates that the decryption process exhibits a computational complexity comparable to that of the encryption process.

To systematically evaluate algorithmic efficiency, the experiment randomly selected 50 RGB-D images from each of the SUNRGBD and Scenes datasets (with sizes 427 × 561 and 480 × 640, respectively), forming a test set of 100 samples. The theoretical analysis established a quantitative relationship between algorithm execution time and input data scale, providing a theoretical foundation for performance optimization. Table 9 presents the average execution times for encryption and decryption operations, with experimental data comprehensively validating the algorithm’s time complexity characteristics in practical scenarios.

### 5.9. Comparison with Other Algorithms

Since the proposed RGB-D encryption scheme essentially operates within a multimodal image encryption framework, we conducted comparisons with State-of-the-Art chaos-based RGB-D encryption algorithms and some multi-image and color image encryption methods. It should be noted that the image datasets used in different studies are not identical. Therefore, average information entropy, absolute correlation coefficient, NPCR, UACI, running time, and throughput are adopted as the primary evaluation metrics. These indicators provide a practical basis for comparison, although differences in datasets, implementations, hardware, and software environments prevent a strictly controlled cross-platform comparison. The detailed results are summarized in Table 10, Table 11 and Table 12, which provide a quantitative comparison of the selected security and computational performance metrics.

As reported in Table 10, the proposed scheme provides a key space of 10^303^ ≈ 2^1007^, which is larger than those of the compared algorithms. This large key space is mainly attributed to the incorporation of the 4D-ICTM chaotic system, whose high-dimensional chaotic dynamics increase the complexity of the encryption process. Such a theoretical key-space size indicates a high search complexity under the assumed parameter precision, although the effective key space may be smaller in finite-precision implementations.

The statistical security evaluation results in Table 11 show that the proposed method outperforms most comparative algorithms in key metrics: information entropy (7.9998), pixel correlation coefficient (<0.005), NPCR (99.6053%), and UACI (33.4682%). The information entropy approaching the ideal value of 8 is ascribed to the high-dimensional random sequences generated by the 4D-ICTM chaotic system, combined with a dual-domain mixing mechanism through hypercomplex frequency-domain transformation. This effectively suppresses the statistical signatures of the original image. Additionally, the sensitivity of 4D-ICTM to initial conditions and the diffusion characteristics of QGT in the frequency domain induce an avalanche effect in response to small plaintext variations. By integrating these features, the method combines statistical uniformity, low adjacent-pixel correlation, and sensitivity to small plaintext changes in the tested experiments.

Encryption efficiency is a crucial criterion for assessing the practicality of image cryptosystems. While encryption time is commonly used to evaluate processing speed, it can vary significantly across different operating environments. Moreover, existing studies on time efficiency often lack consistency in image size or quantity, which complicates meaningful comparison. To overcome this limitation, we adopt encryption throughput and number of cycles per byte as evaluation metrics, thereby enabling a more reliable and comprehensive assessment of time efficiency. Their computation is defined as follows [60]:

(34) $$ET=\frac{TotalImageData(Bit)}{Encryption_{Time}(Second)}$$

(35) $$NCPB=\frac{CPU_{Speed}(Hertz)}{ET(Byte/Second)}$$

Equation (34) defines the encryption throughput, expressed in Mbps (1 Mbps = 10^6^ bits), which characterizes the processing rate of the encryption algorithm. Equation (35) introduces the normalized CPU cycles per byte, representing the average number of CPU cycles required to encrypt each byte. Since the denominator of Equation (35) converts ET into a byte-per-second form, *NCPB* can be further written as $NCPB=8\times {CPU}_{speed}/ET$, indicating that *NCPB* is inversely proportional to *ET*. Thus, *ET* and *NCPB* provide complementary measures of processing efficiency, with higher *ET* and lower *NCPB* values indicating better performance [60].

Table 12 evaluates the computational time of the proposed scheme against several representative multi-image and color image encryption algorithms. The results provide a quantitative comparison of computational efficiency. Specifically, the *ET* of the proposed scheme reaches 43.3271 Mbps, which is more than three times higher than the best result achieved by existing methods (13.6105 Mbps). In addition, the *NCPB* is only 406 cycles/byte, which is at most one-fourth of the minimum *NCPB* among the compared algorithms (1881 cycles/byte). These findings indicate that the proposed algorithm achieves higher measured processing throughput with fewer CPU cycles under the tested conditions. The results therefore provide a direct benchmark for the computational efficiency of the proposed encryption scheme.

The contribution of the main components is further examined through component-level controlled comparisons. For the QGT, the proposed direct formulation is compared with the conventional dual-QFT implementation under the same quaternion input and experimental environment, showing numerical equivalence while reducing the execution time. The dynamical and statistical analyses in Section 2 similarly verify the suitability of 4D-ICTM for chaotic sequence generation. At the complete-scheme level, Table 11 and Table 12 provide security and efficiency comparisons with representative existing methods. For this architecture, the QGT provides the quaternion-domain operation required for the unified representation of the four RGB-D components. Replacing it with a conventional single-channel transform would therefore change the underlying processing framework rather than isolate the contribution of a single component.

## 6. Discussion

The proposed scheme combines the 4D-ICTM hyperchaotic system with the reformulated QGT to jointly process the four components of the RGB-D data. The experimental results indicate that this combination provides diffusion, permutation, and spatial-correlation reduction while maintaining the measured computational efficiency. The proposed QGT also achieves numerical consistency with the conventional implementation and reduces the computational cost, supporting its use in the overall encryption framework.

The security evaluation is based on experimental analyses, including key sensitivity, statistical properties, correlation, floating frequency, graphic autocorrelation, and decryption error. These results demonstrate resistance to the tested attacks. However, they should not be interpreted as a formal proof of cryptographic security. In particular, effective entropy and security against advanced cryptanalytic or side-channel attacks require further theoretical and experimental investigation.

The encryption efficiency is evaluated against representative multi-image and color-image encryption schemes because comparable RGB-D encryption methods are still limited. Therefore, the reported ET and NCPB values provide a practical reference rather than a strict cross-platform benchmark, since implementation environments, hardware, and programming platforms may differ.

For practical deployment, the proposed scheme may also require further optimization for resource-constrained devices. In addition, the error-propagation characteristics observed under noise and data loss attacks indicate that the trade-off between encryption security and transmission robustness should be considered in specific applications. Future work will focus on improving implementation efficiency, evaluating the scheme on different hardware platforms, further investigating its security under more advanced attack models, assessing its robustness under more severe channel distortions, and exploring its integration with deep learning [64] for intelligent image encryption.

## 7. Conclusions

This study develops an RGB-D image encryption framework utilizing a newly constructed 4D-ICTM hyperchaotic system and a reformulated quaternion gyrator transform (QGT). To accommodate the heterogeneous nature of RGB color information and 16-bit depth data, a dedicated preprocessing strategy is designed to reorganize raw RGB-D inputs into four standardized 8-bit components. Such transformation enables unified quaternion-domain representation and joint processing of multimodal image data. The 4D-ICTM system produces chaotic sequences for pixel permutation and value diffusion, while the reformulated QGT provides a numerically consistent yet computationally efficient quaternion transformation module for the overall encryption pipeline.

Experimental validations demonstrate that the proposed method achieves satisfactory statistical uniformity, high sensitivity to secret-key variations, and ciphertext sensitivity to differential changes. It also preserves reliable decryption performance under noise contamination and partial data loss during transmission. Compared with the conventional QGT implementation, the reformulated version preserves numerical consistency while reducing the measured execution time by approximately 21.8%. The proposed scheme also achieves an encryption throughput of 43.3271 Mbps and 406 cycles/byte under the tested experimental conditions.

The reported security performance is based on experimental evaluations under specific test conditions and should not be regarded as a formal proof of cryptographic security. Further investigation is required to analyze the effective key entropy, evaluate security against advanced cryptanalytic and side-channel attacks, and assess performance across different hardware and implementation environments. Moreover, robustness under more severe channel distortion and hardware adaptation for resource-constrained devices will be further explored.

## Figures and Tables

**Figure 1:**
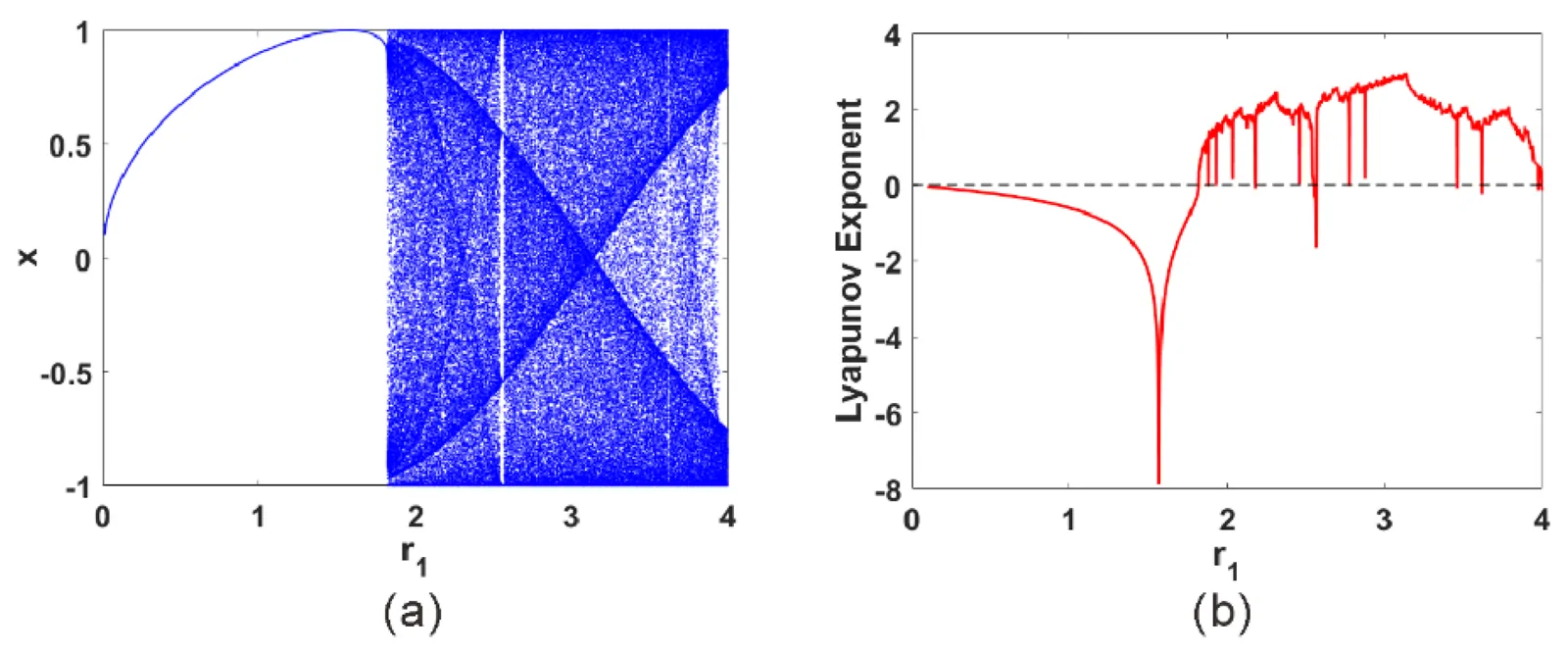
ICMIC map. (**a**) bifurcation diagram; (**b**) Lyapunov exponents.

**Figure 2:**
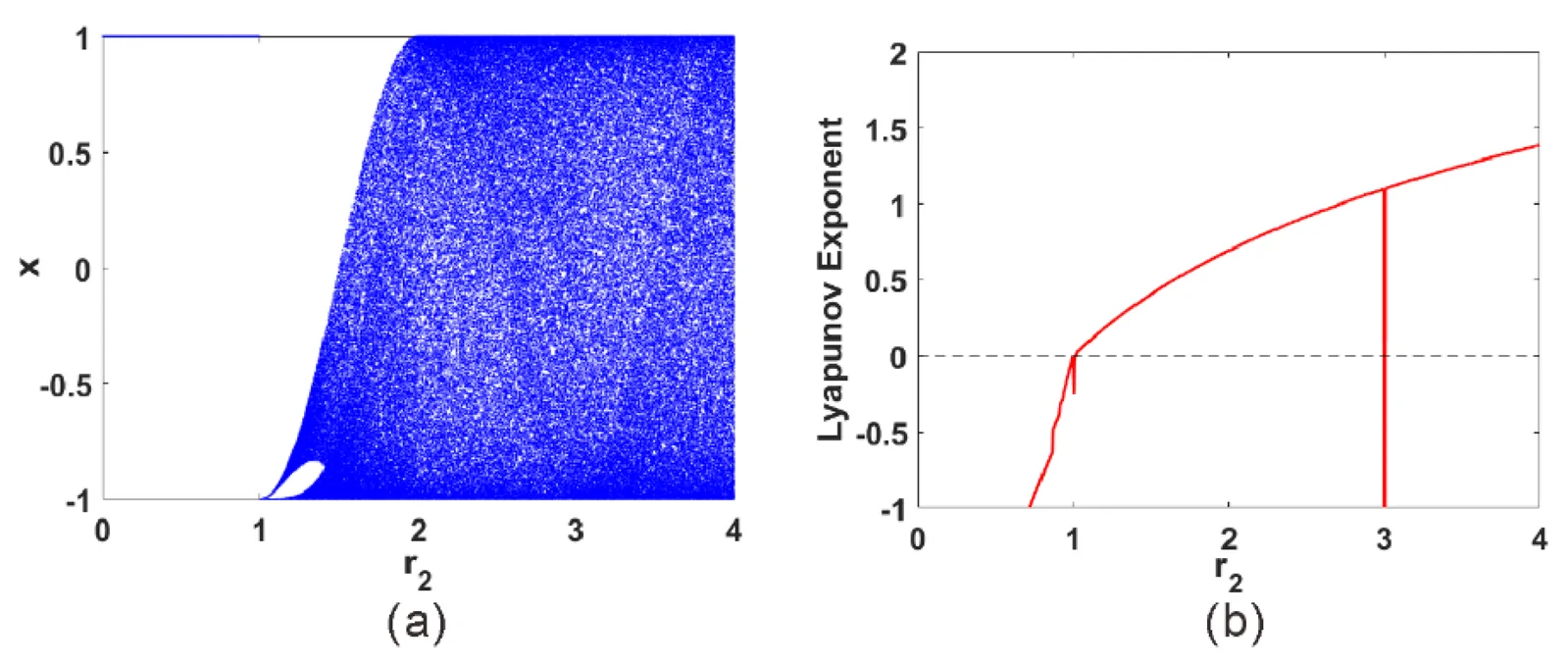
Chebyshev map. (**a**) bifurcation diagram; (**b**) Lyapunov exponents.

**Figure 3:**
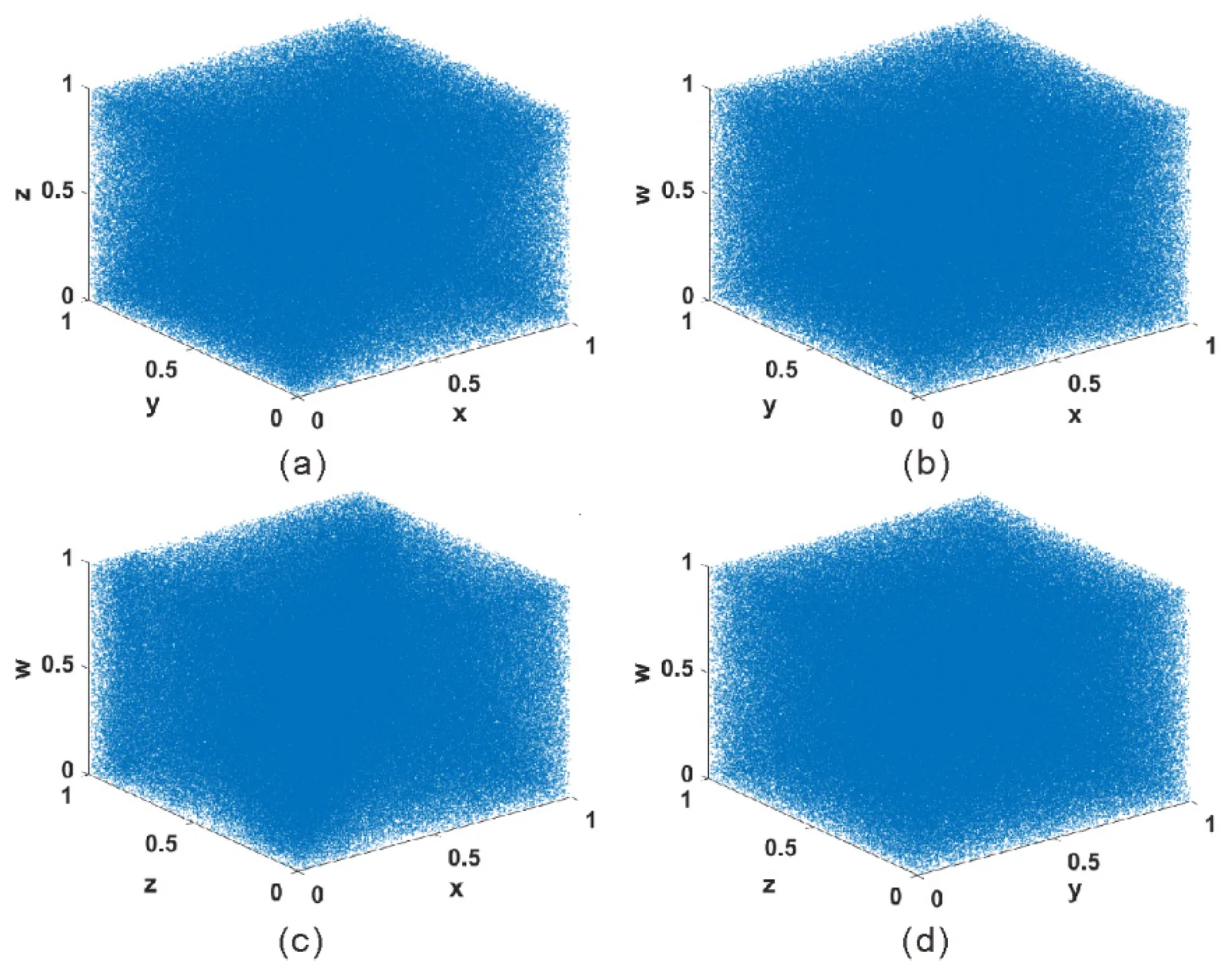
Attractor phase diagram of the 4D-ICTM. (**a**) *x*-*y*-*z*; (**b**) *x*-*y*-*w*; (**c**) *x*-*z*-*w*; (**d**) *y*-*z*-*w*.

**Figure 4:**
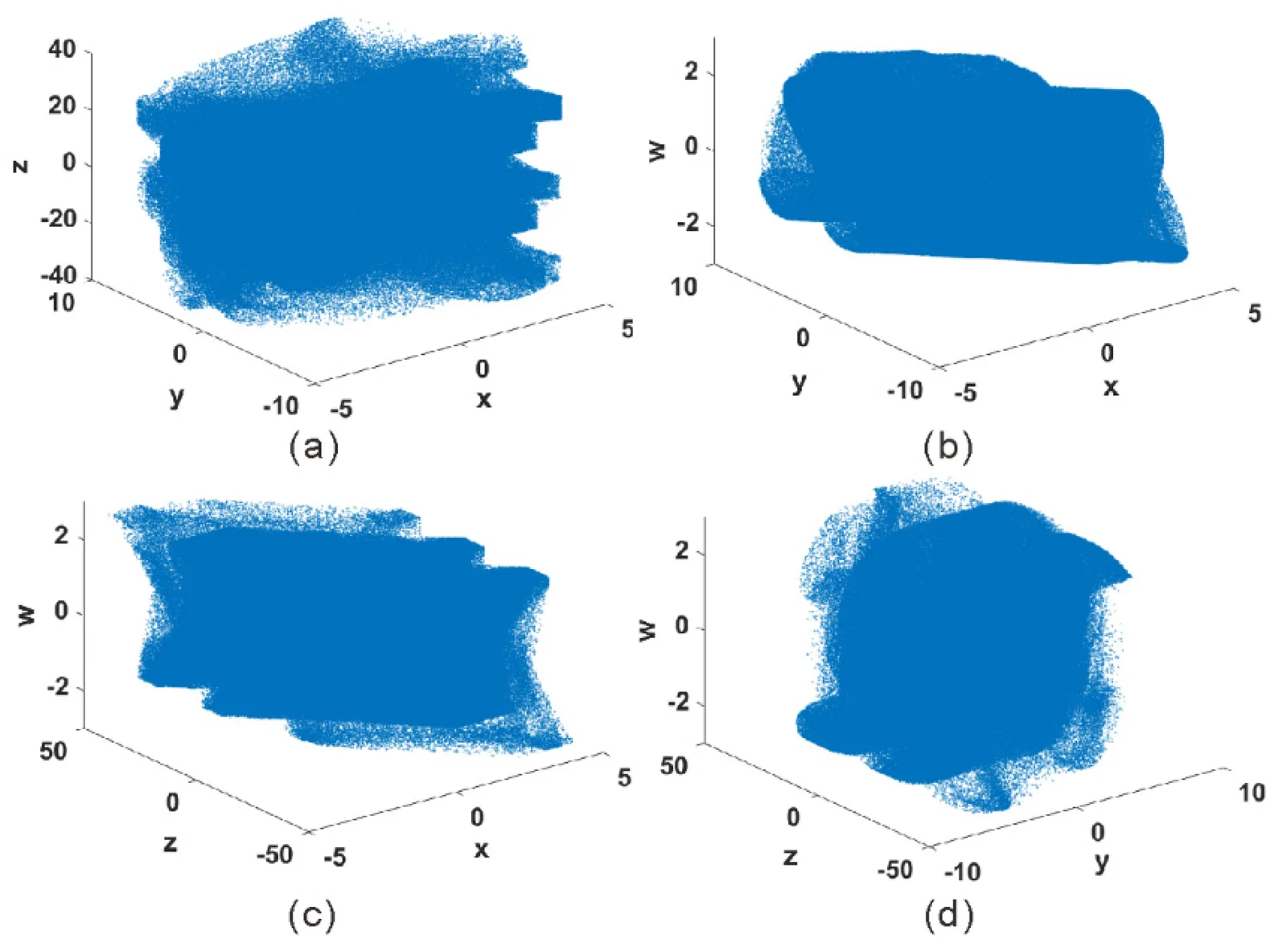
Attractor phase diagram of the 4D chaotic map in [21]. (**a**) *x*-*y*-*z*; (**b**) *x*-*y*-*w*; (**c**) *x*-*z*-*w*; (**d**) *y*-*z*-*w*.

**Figure 7:**
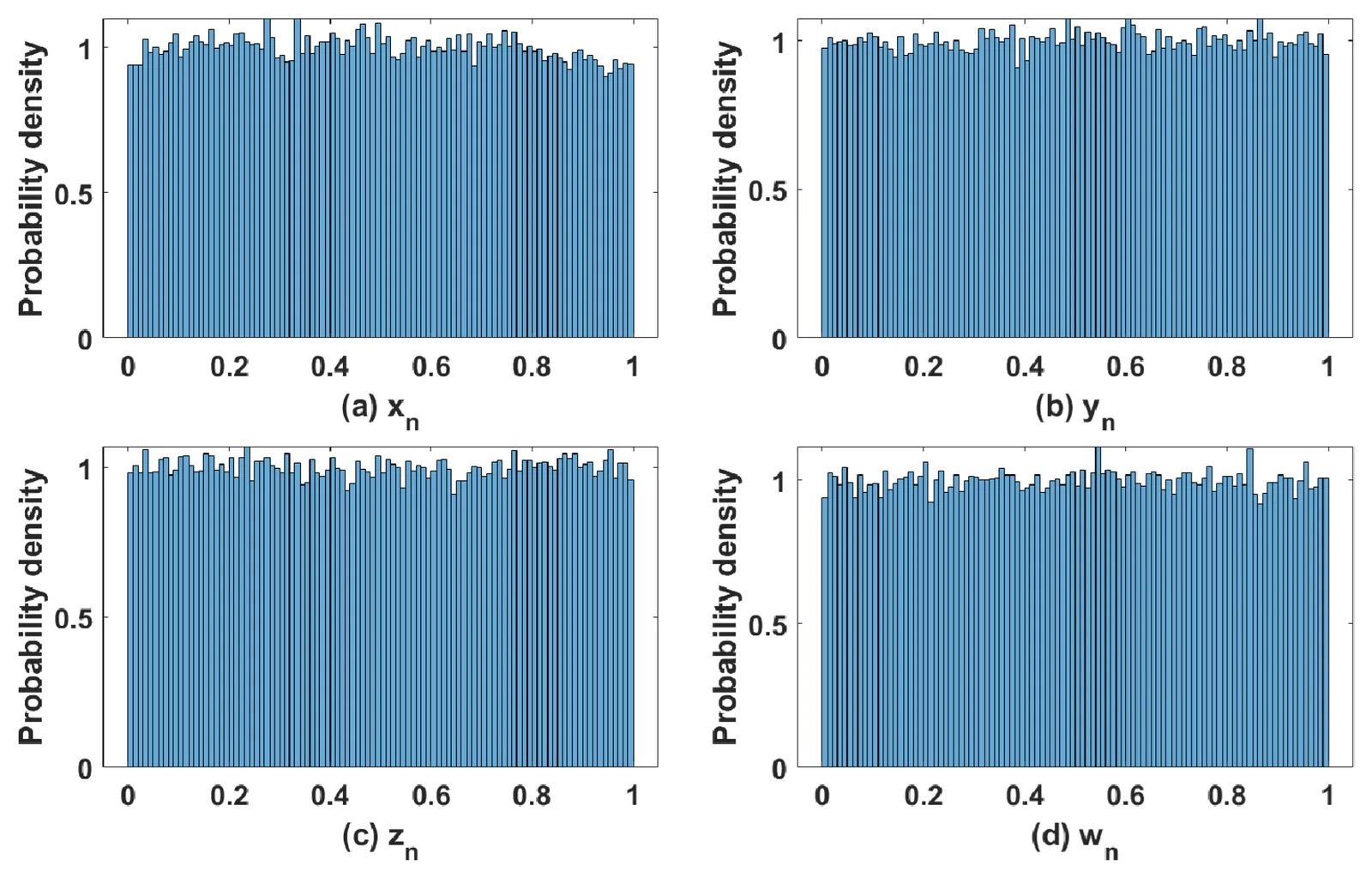
Histograms of the four state variables of the 4D-ICTM. (**a**) $x_{n}$; (**b**) $y_{n}$; (**c**) $z_{n}$; (**d**) $w_{n}$.

**Figure 8:**
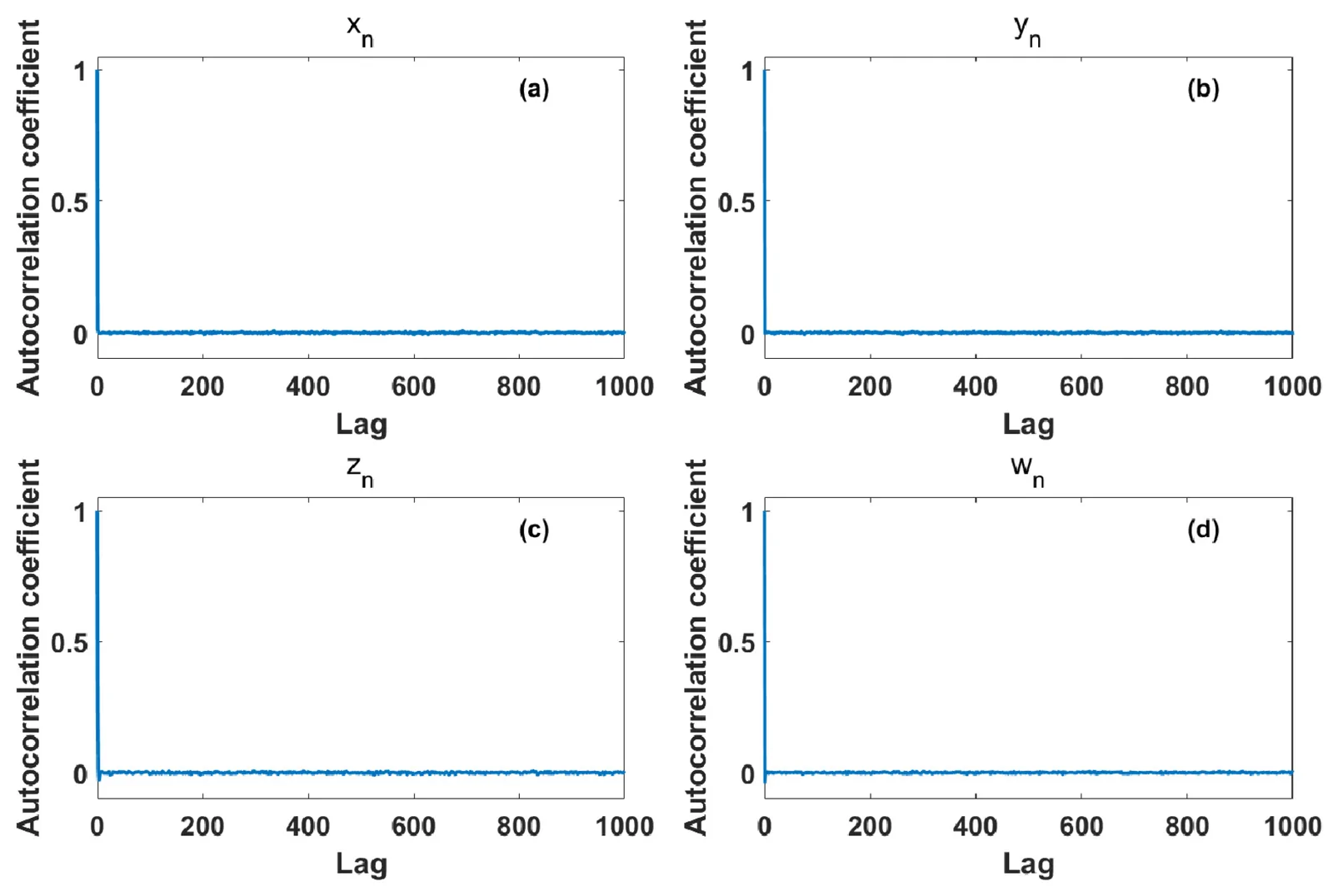
Autocorrelation of the four state variables of the 4D-ICTM. (**a**) $x_{n}$; (**b**) $y_{n}$; (**c**) $z_{n}$; (**d**) $w_{n}$.

**Figure 9:**
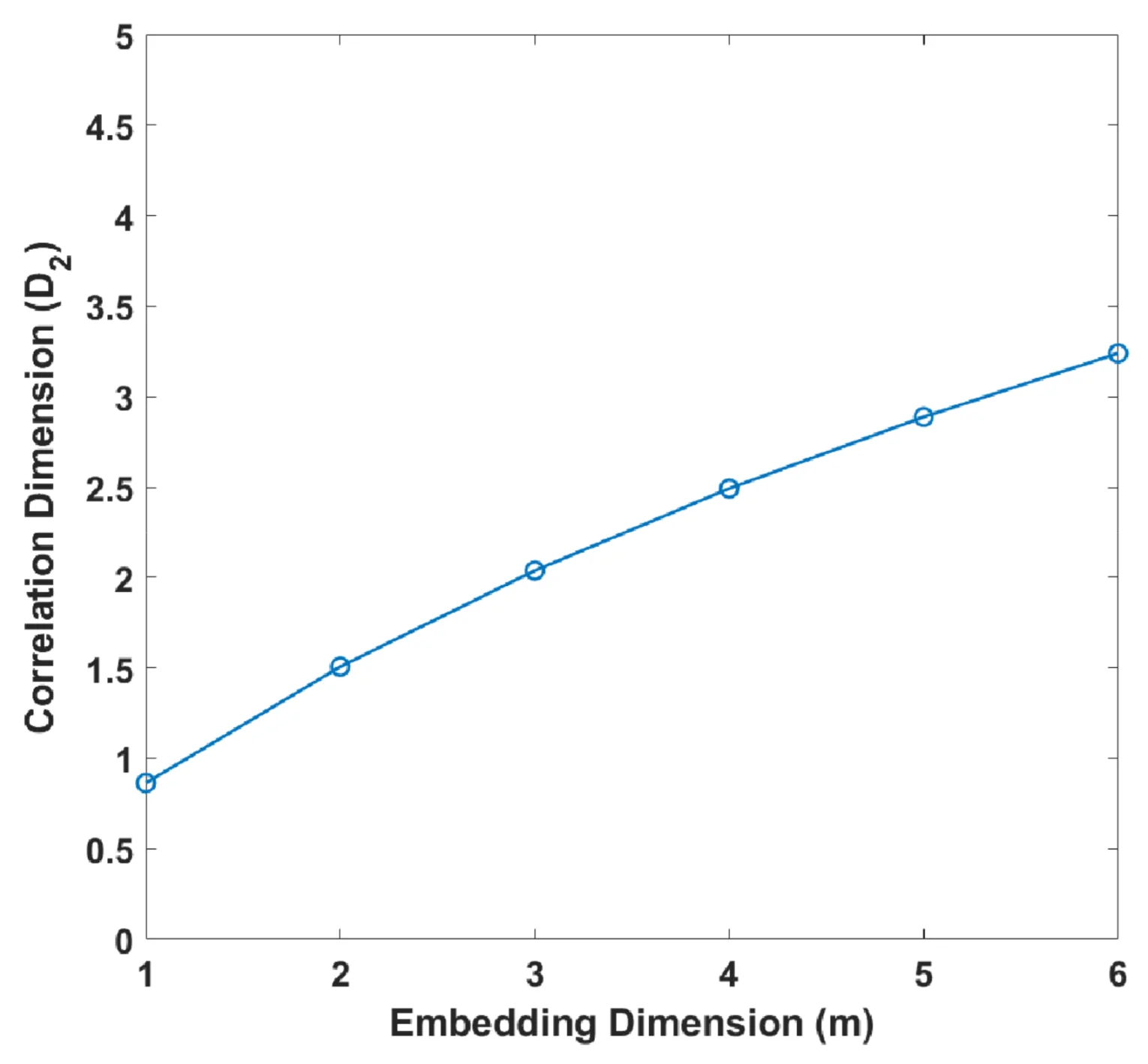
Correlation dimension analysis of the 4D-ICTM using the Grassberger-Procaccia algorithm.

**Figure 10:**
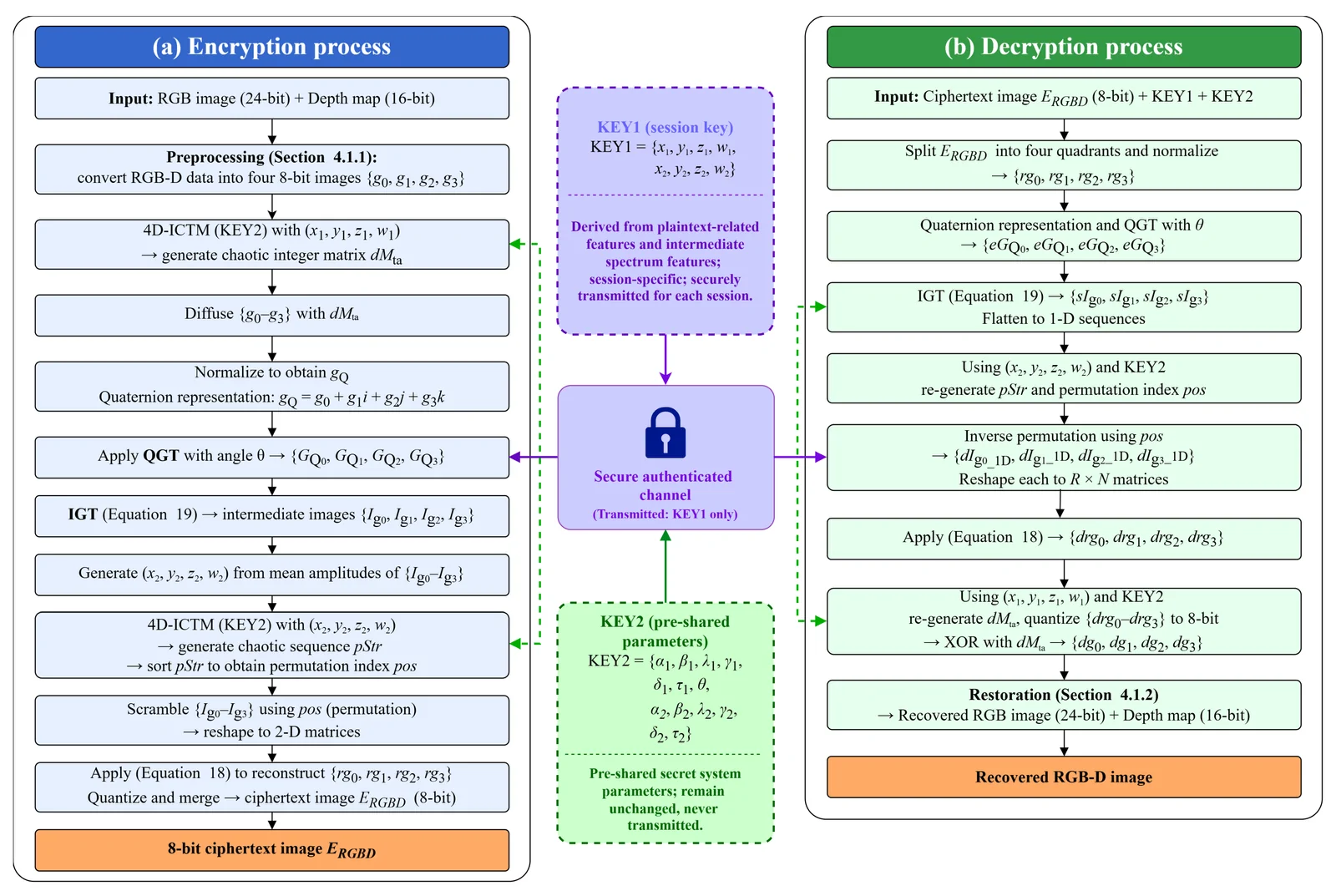
Block diagrams of the proposed RGB-D image encryption and decryption processes.

**Figure 11:**
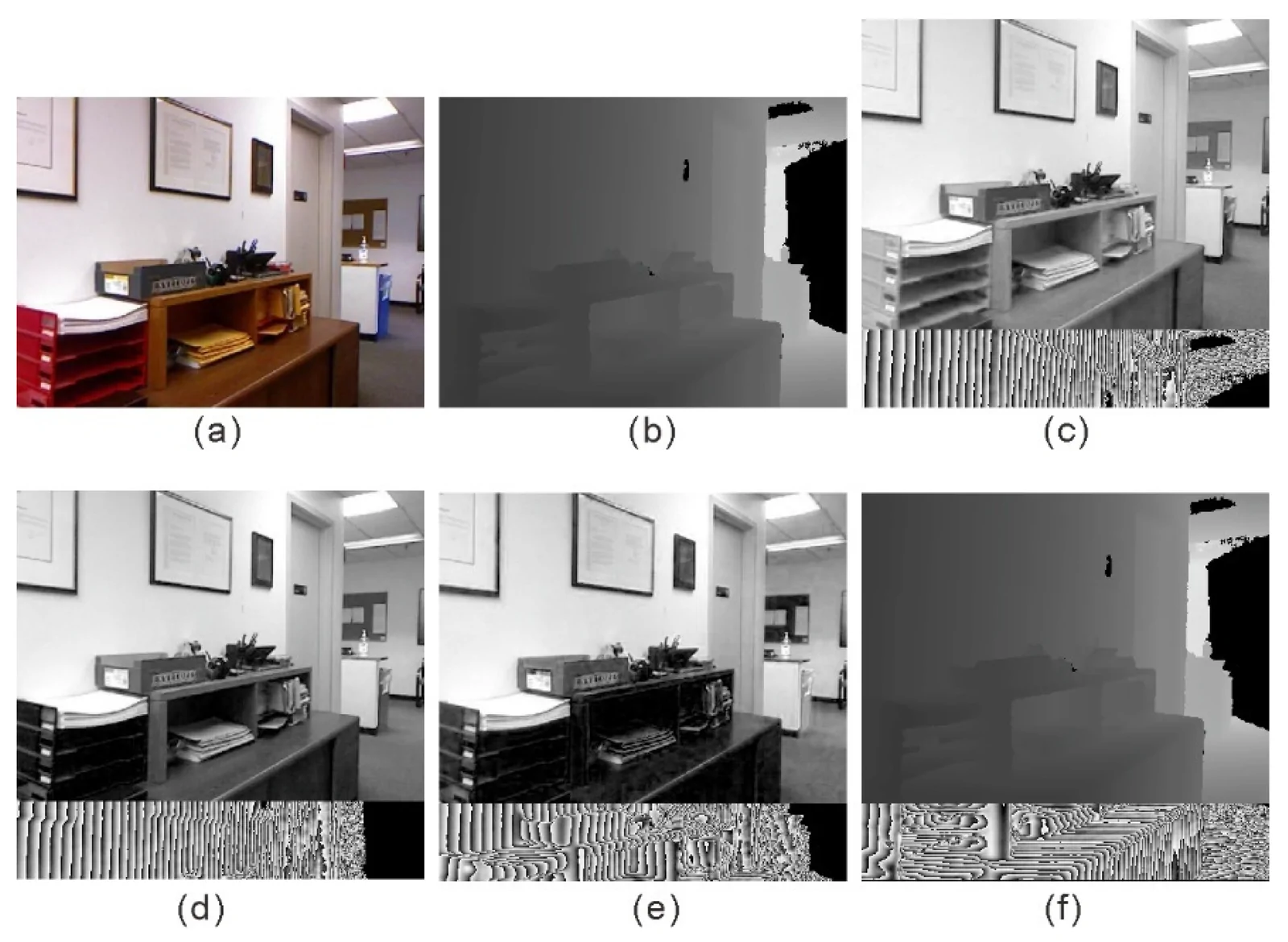
Preprocessing of RGB-D image NYU0009. (**a**) 24-bit RGB component (427 × 561); (**b**) 16-bit depth map (427 × 561); (**c**) 8-bit merged image *g*_0_ (534 × 561); (**d**) 8-bit *g*_1_ (534 × 561); (**e**) 8-bit *g*_2_ (534 × 561); (**f**) 8-bit *g*_3_ (534 × 561).

**Figure 12:**
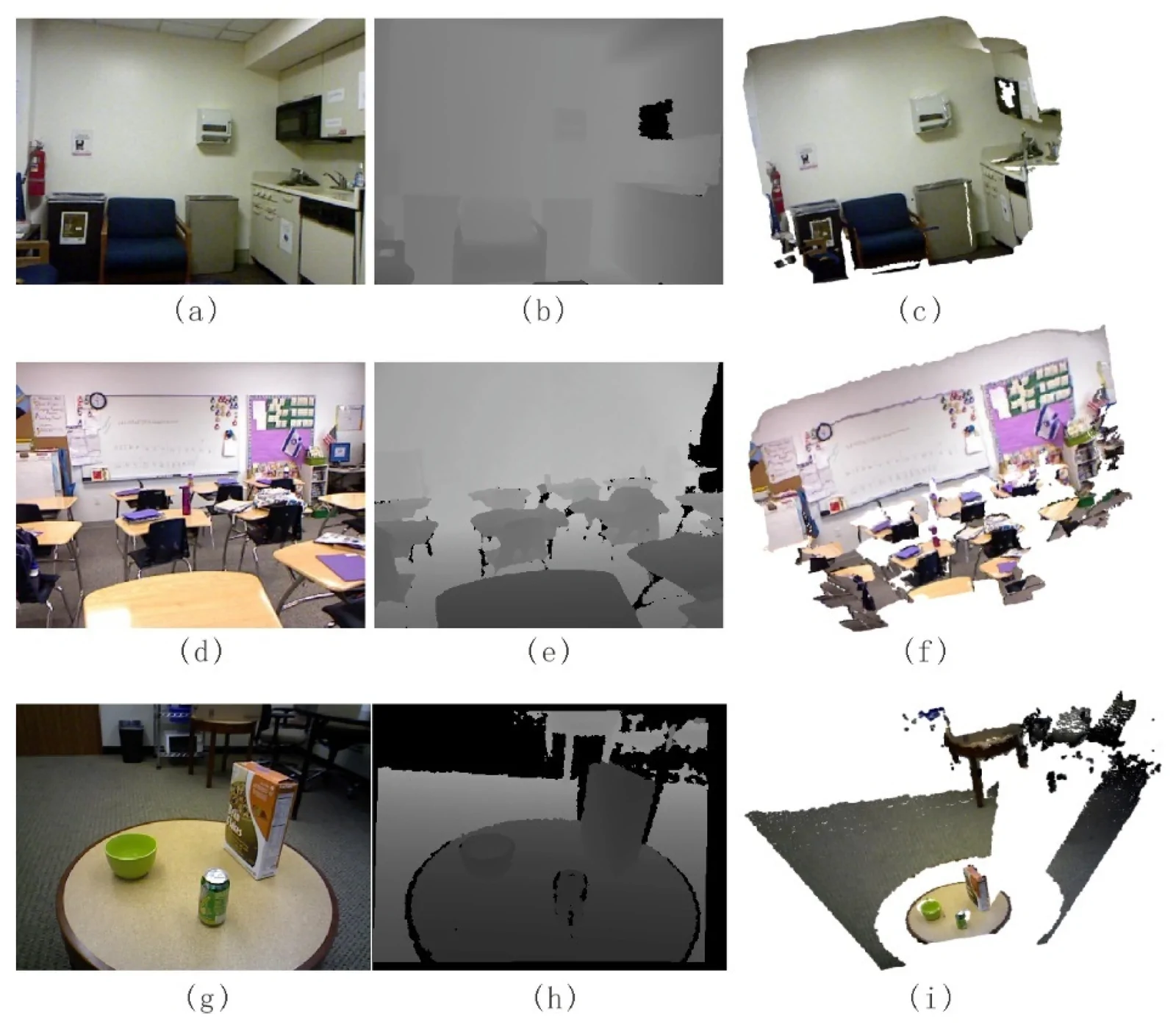
Original RGB-D images. (**a**) RGB image of NYU0001 (427 × 561); (**b**) depth map of NYU0001 (427 × 561); (**c**) 3D point cloud visualization of NYU0001 (azimuth 10°, elevation −80°, 875 × 656); (**d**) RGB image of NYU0300 (427 × 561); (**e**) depth map of NYU0300 (427 × 561); (**f**) 3D point cloud visualization of NYU0300 (azimuth 10°, elevation −80°, 875 × 656); (**g**) RGB image of 00666 (480 × 640); (**h**) depth map of 00666 (480 × 640); (**i**) 3D point cloud visualization of 00666 (azimuth 10°, elevation −80°, 875 × 656).

**Figure 13:**
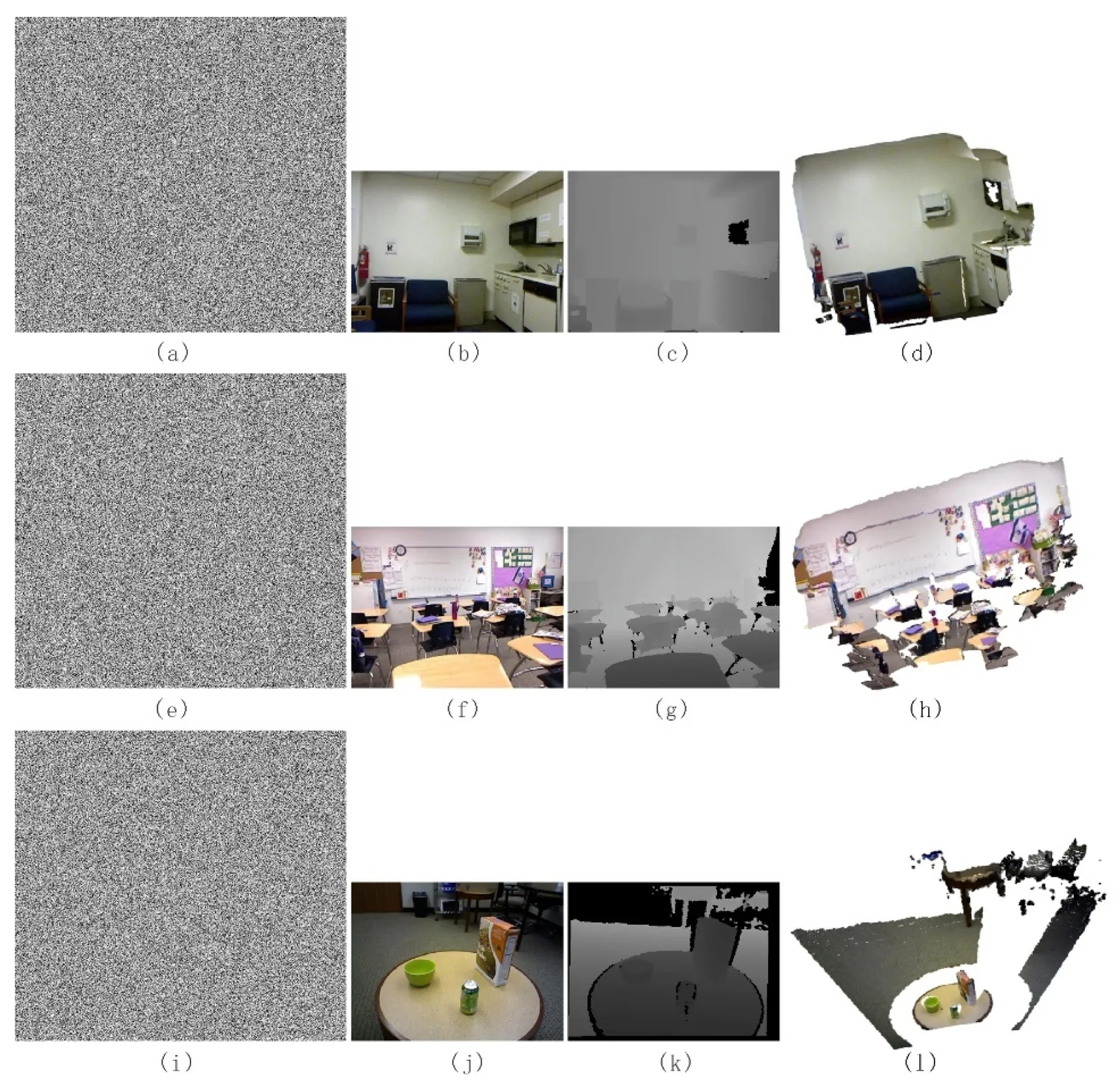
Encryption and decryption results of the proposed image cryptography. (**a**) Encrypted image of NYU0001 (1068 × 1122); (**b**) decrypted RGB image of NYU0001 (427 × 561); (**c**) decrypted depth map of NYU0001 (427 × 561); (**d**) 3D point cloud visualization of decrypted NYU0001 (875 × 656); (**e**) Encrypted image of NYU0300 (1068 × 1122); (**f**) decrypted RGB image of NYU0300 (427 × 561); (**g**) decrypted depth map of NYU0300 (427 × 561); (**h**) 3D point cloud visualization of decrypted NYU0300 (875 × 656); (**i**) Encrypted image of 00666 (1200 × 1280); (**j**) decrypted RGB image of 00666 (480 × 640); (**k**) decrypted depth map of 00666 (480 × 640); (**l**) 3D point cloud visualization of decrypted 0066 (875 × 656).

**Figure 14:**
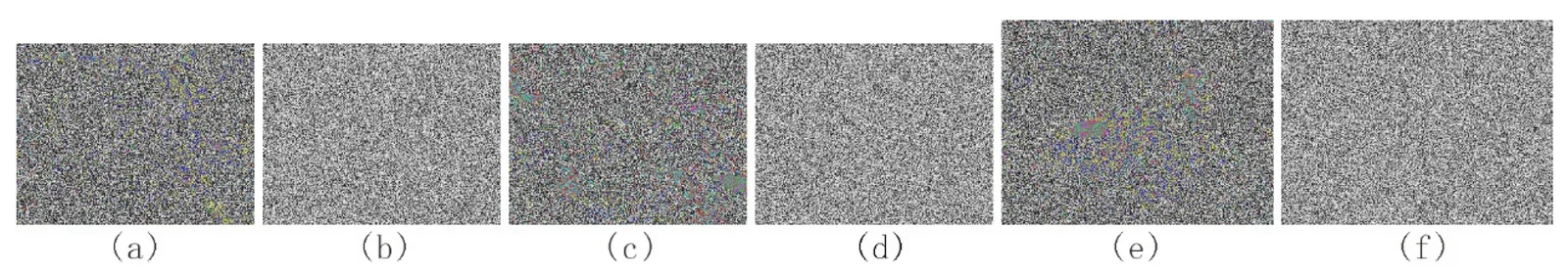
Decrypted RGB-D image with *x*1 = *x*1 + 10^−15^. (**a**,**b**) NYU0001 (RGB, depth); (**c**,**d**) NYU0300 (RGB, depth); (**e**,**f**) 00666 (RGB, depth).

**Figure 15:**
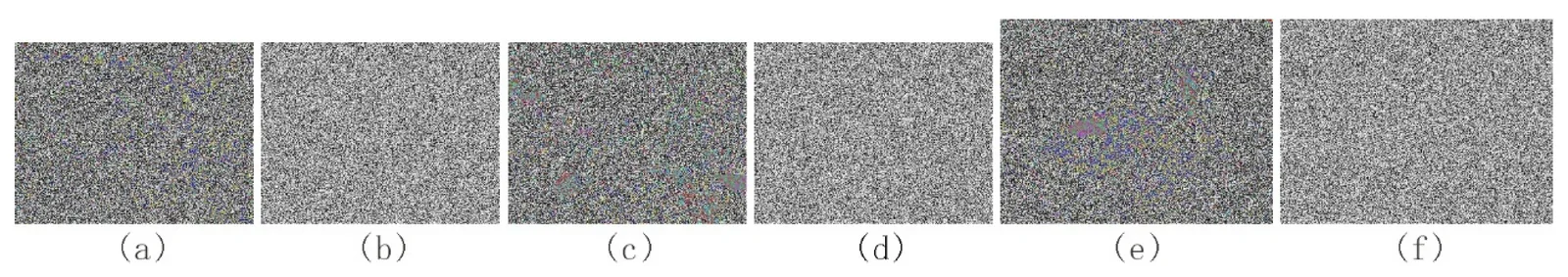
Decrypted RGB-D image with *λ*1 = *λ*1 + 10^−15^. (**a**,**b**) NYU0001 (RGB, depth); (**c**,**d**) NYU0300 (RGB, depth); (**e**,**f**) 00666 (RGB, depth).

**Figure 16:**
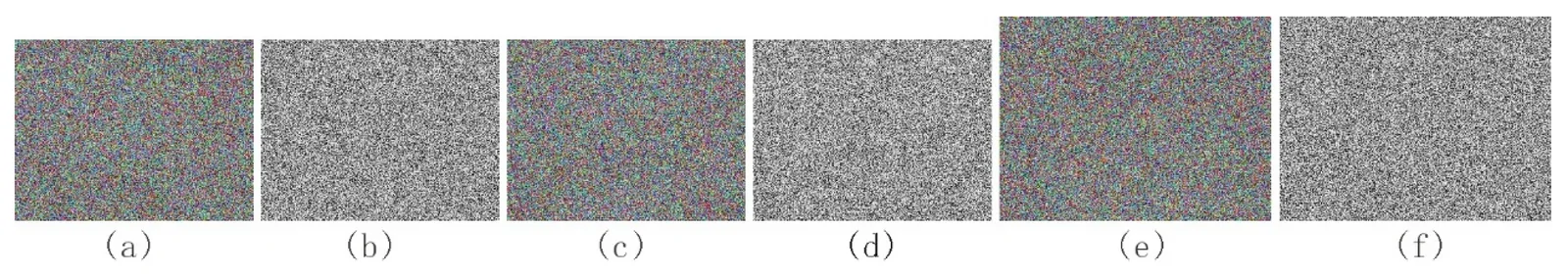
Decrypted RGB-D image with *θ* = *θ* + 10^−3^. (**a**,**b**) NYU0001 (RGB, depth); (**c**,**d**) NYU0300 (RGB, depth); (**e**,**f**) 00666 (RGB, depth).

**Figure 17:**
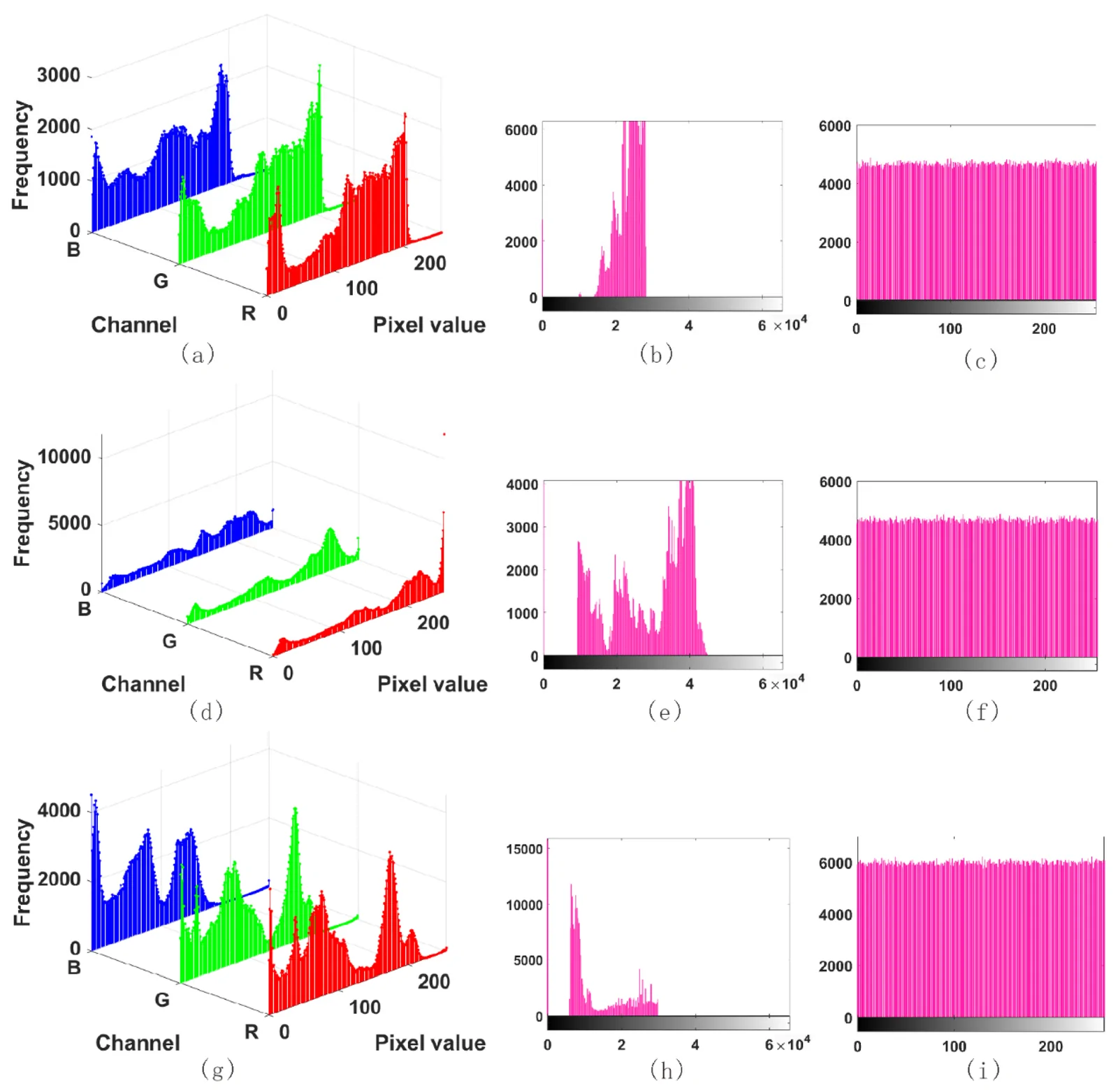
Histograms of three plaintext and encrypted RGB-D images. (**a**–**c**) Red, green, blue channels of the color component, depth map, and encrypted image of NYU0001. (**d**–**f**) Red, green, blue channels of the color component, depth map, and encrypted image of NYU0300. (**g**–**i**) Red, green, blue channels of the color component, depth map, and encrypted image of 00666.

**Figure 18:**
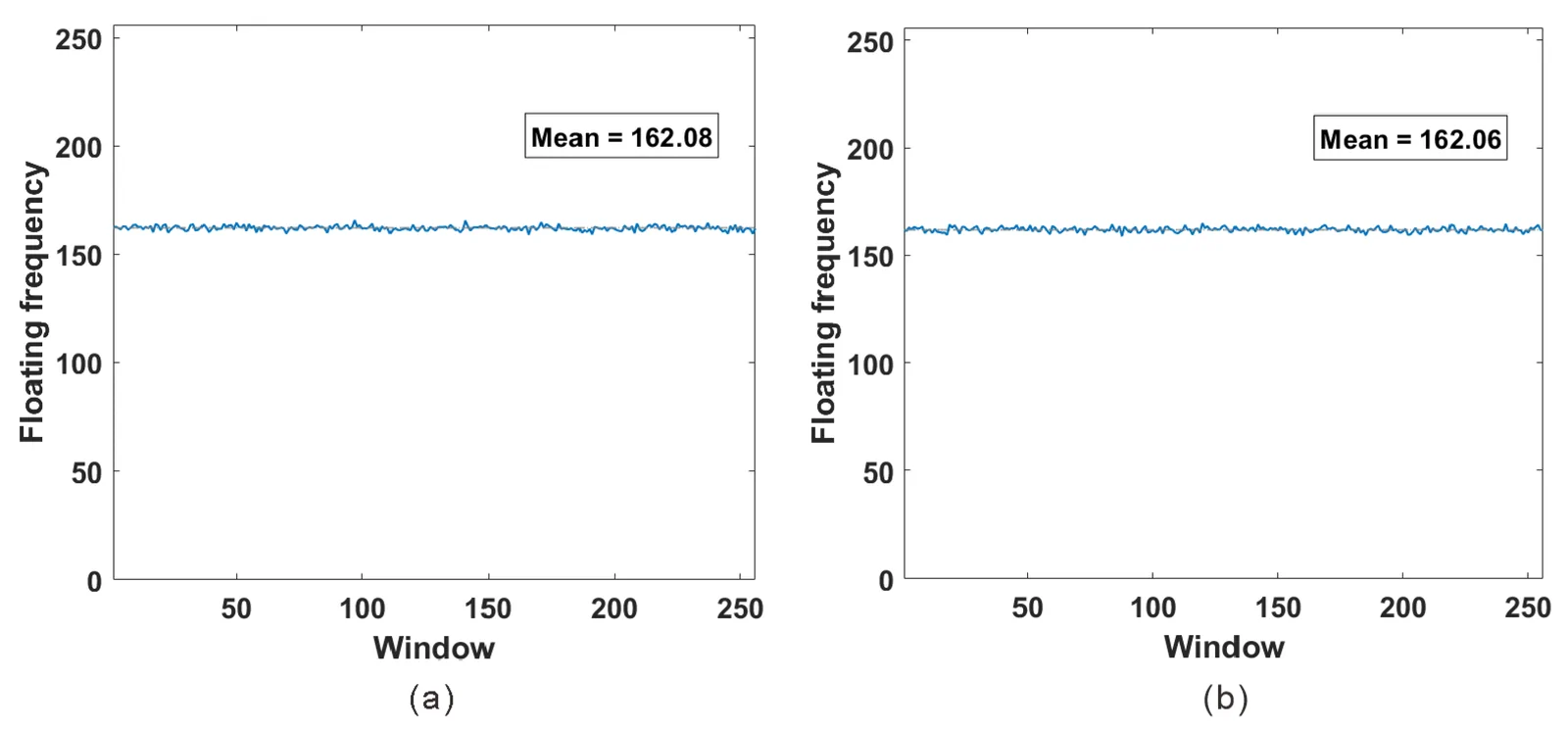
Floating frequency assessment for the NYU0001 ciphertext: (**a**) row-wise floating frequency with its average; (**b**) column-wise floating frequency with its average.

**Figure 19:**
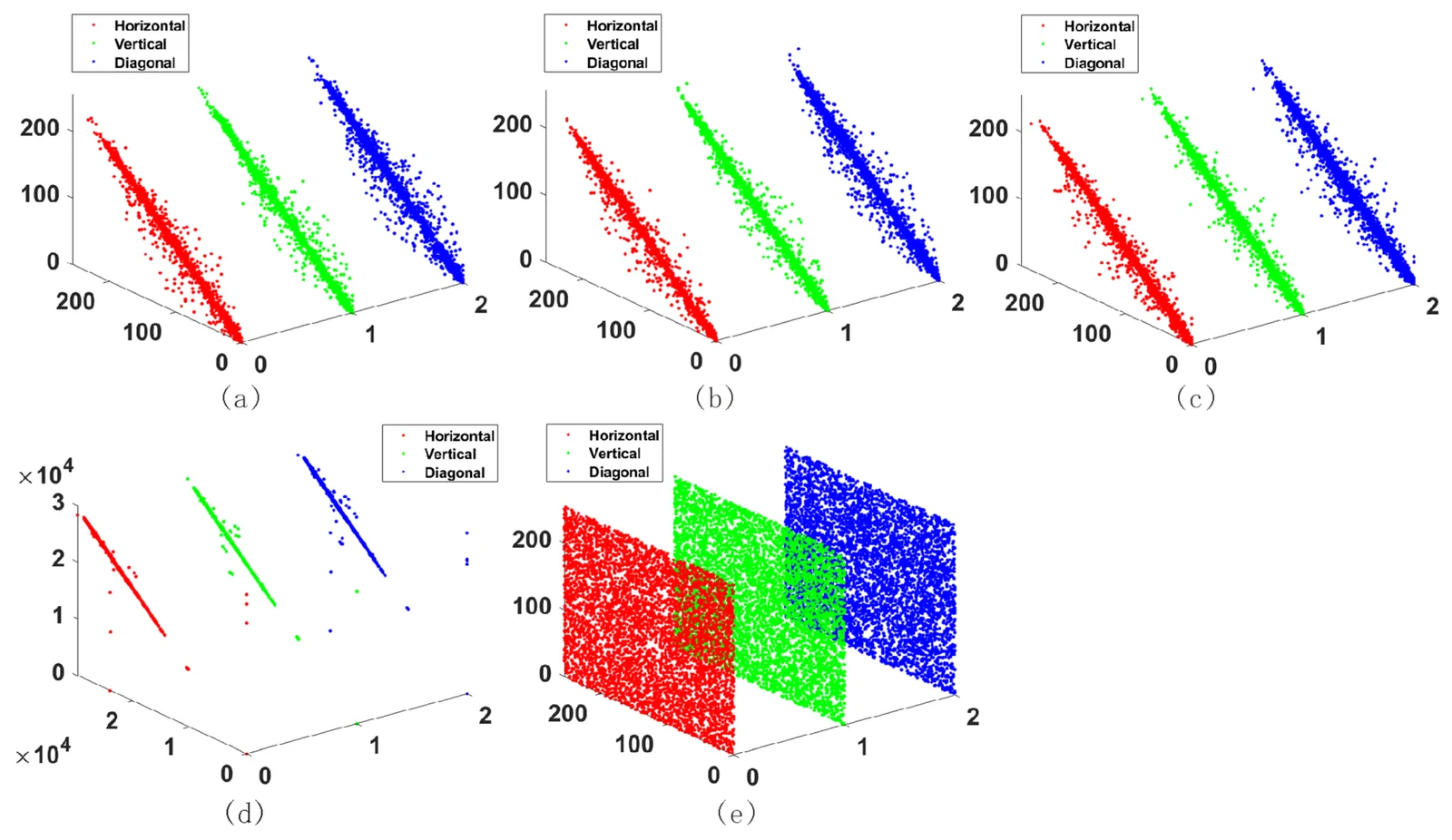
Correlation of adjacent pixels for the plain RGB-D image “NYU0001” and its ciphertext. (**a**) red channel; (**b**) green channel; (**c**) blue channel; (**d**) depth map; (**e**) encrypted image.

**Figure 20:**
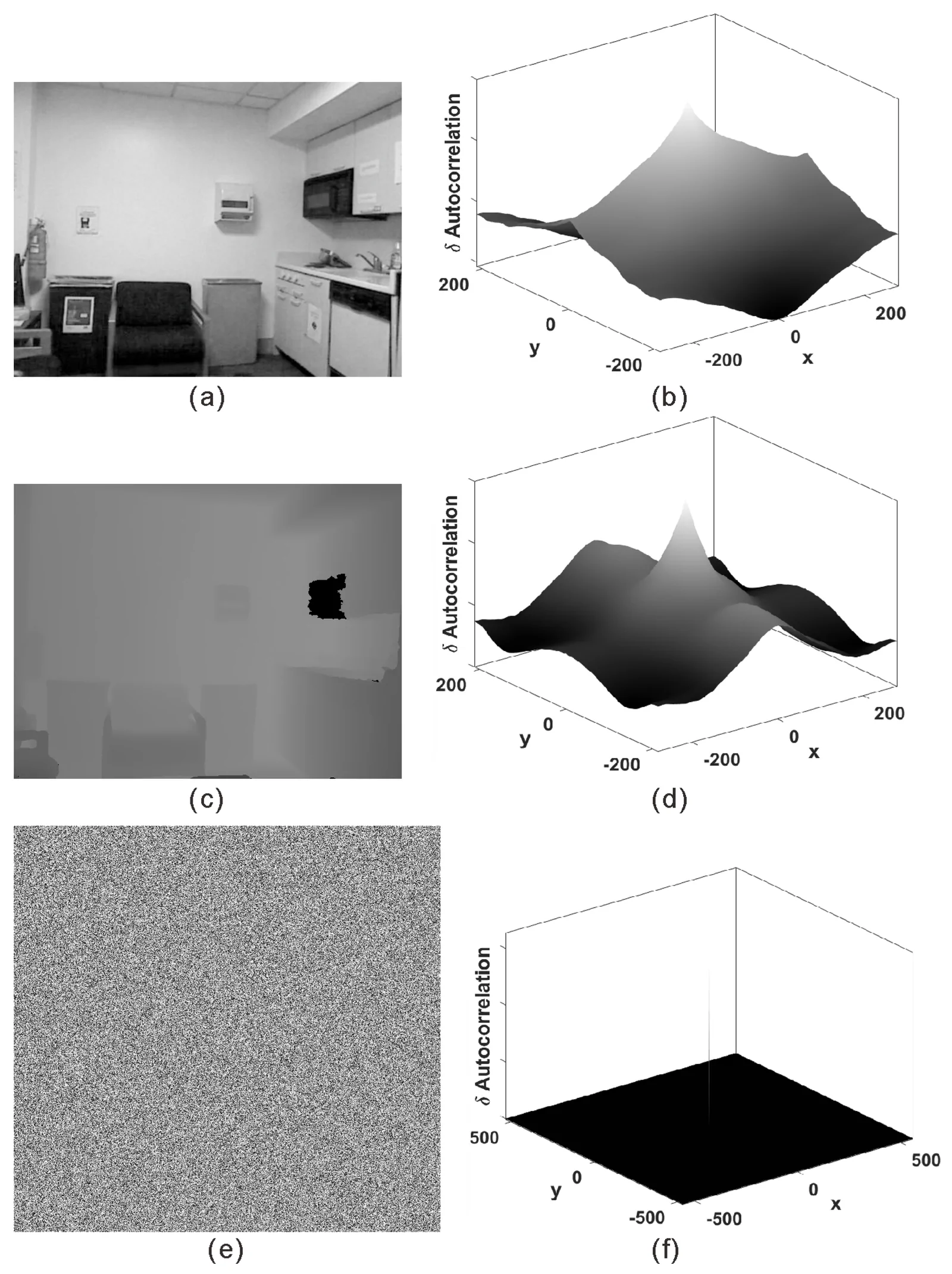
Graphic autocorrelation analysis of NYU0001: (**a**) R channel, (**b**) its autocorrelation, (**c**) depth map, (**d**) its autocorrelation, (**e**) ciphertext, and (**f**) its autocorrelation.

**Figure 21:**
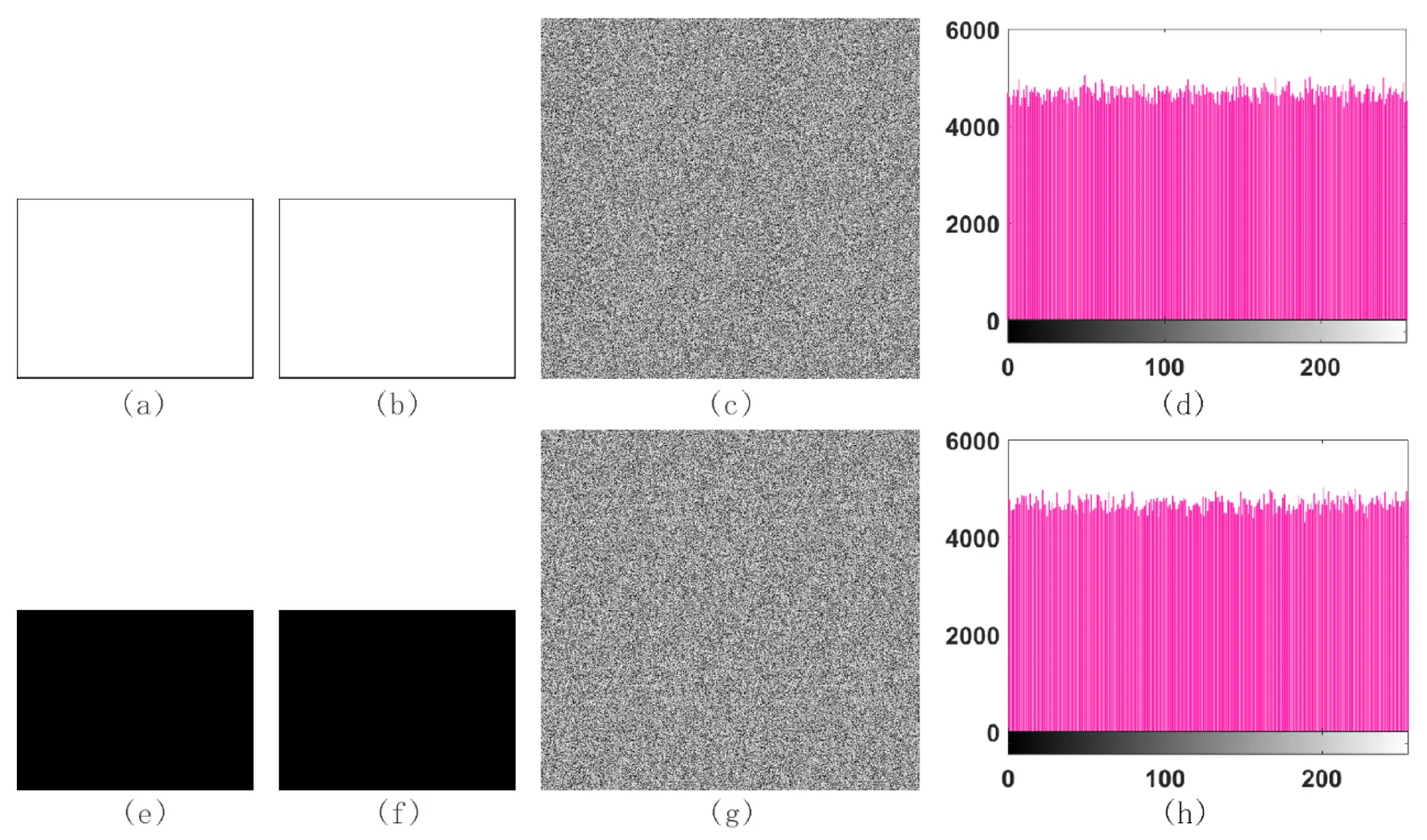
Plaintext attack evaluation. (**a**) all-white RGB image; (**b**) all-white depth map; (**c**) encrypted image of all-white RGB-D image; (**d**) histogram of (**c**); (**e**) all-black RGB image; (**f**) all-black depth map; (**g**) encrypted image of all-black RGB-D image; (**h**) histogram of (**g**).

**Figure 22:**
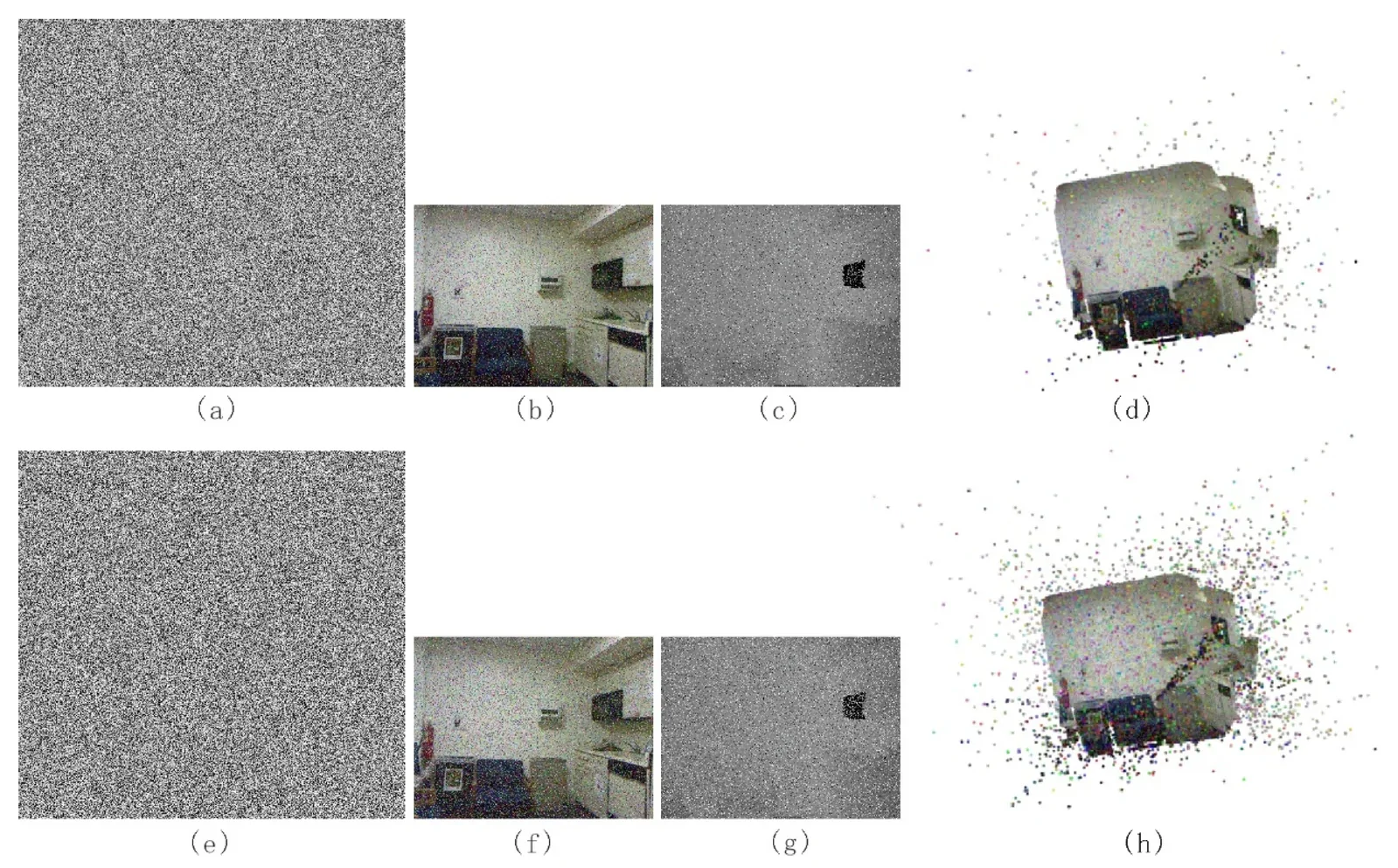
Noise attacks. (**a**) corrupted ciphertext with 20% salt-and-pepper noise; (**b**) decrypted RGB component of (**a**); (**c**) decrypted depth map of (**a**); (**d**) 3D visualization; (**e**) corrupted ciphertext with 30% salt-and-pepper noise; (**f**) decrypted RGB component of (**e**); (**g**) decrypted depth map of (**e**); (**h**) 3D visualization.

**Figure 23:**
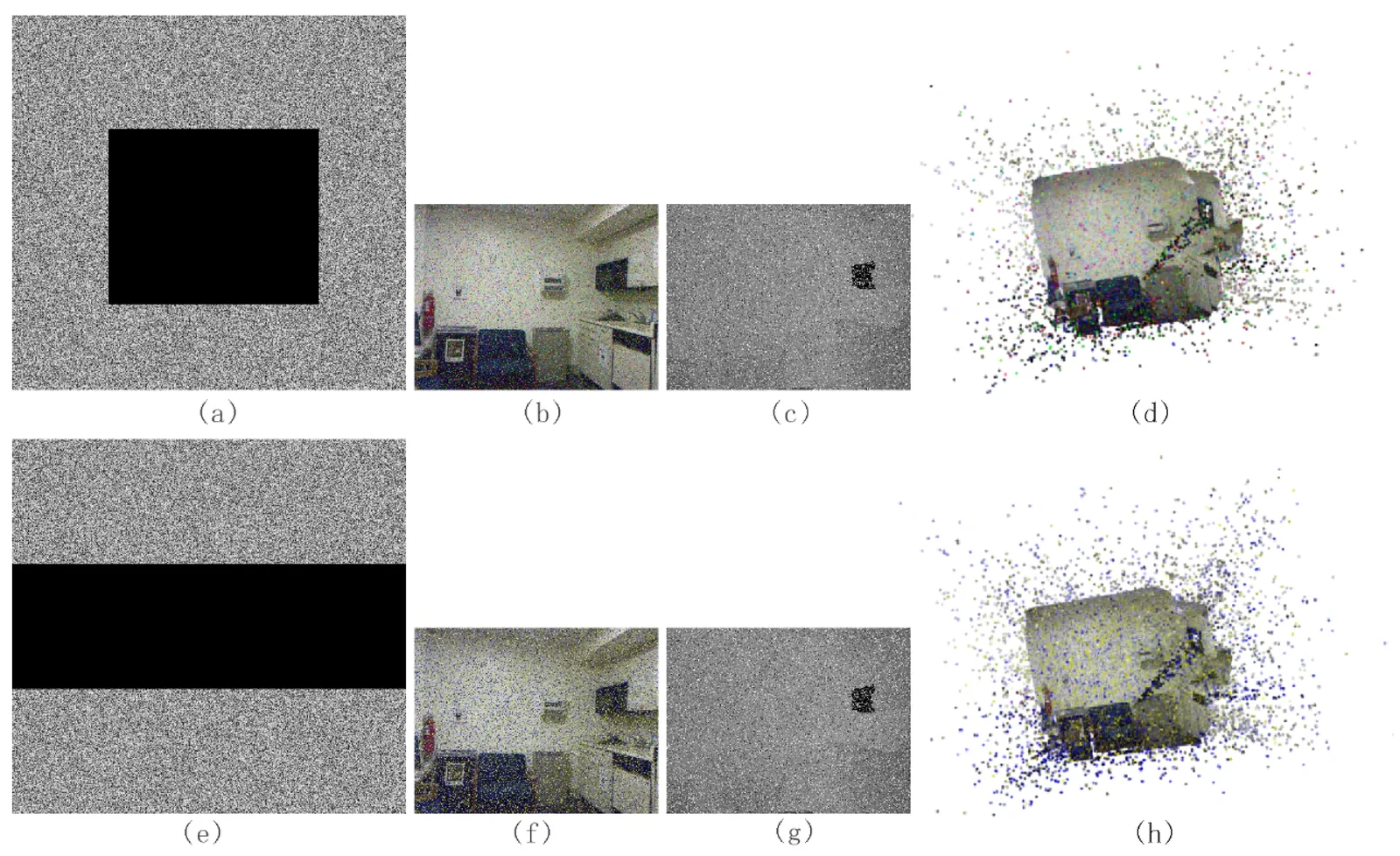
Data loss attacks. (**a**) 1/4 occluded ciphertext; (**b**) decrypted RGB component of (**a**); (**c**) decrypted depth map of (**a**); (**d**) 3D visualization; (**e**) 1/3 occluded ciphertext; (**f**) decrypted RGB component of (**e**); (**g**) decrypted depth map of (**e**); (**h**) 3D visualization.

**Table 1:** NIST test results of the 4D-ICTM system.

Statistical Test	*x*		*y*		*z*		*w*	
	*p*-Value	Pass /Fail	*p*-Value	Pass /Fail	*p*-Value	Pass /Fail	*p*-Value	Pass /Fail
Frequency	0.637119	pass	0.025193	pass	0.437274	pass	0.534146	pass
Block frequency	0.275709	pass	0.739918	pass	0.350485	pass	0.637119	pass
Cumulative sums	0.534146	pass	0.122325	pass	0.066882	pass	0.739918	pass
Runs	0.739918	pass	0.213309	pass	0.834308	pass	0.911413	pass
Longest runs of ones	0.911413	pass	0.275709	pass	0.964295	pass	0.534146	pass
Rank	0.834308	pass	0.534146	pass	0.534146	pass	0.437274	pass
FFT	0.350485	pass	0.437274	pass	0.122325	pass	0.834308	pass
No overlapping templates	0.066882	pass	0.991468	pass	0.090936	pass	0.350485	pass
Overlapping templates	0.048716	pass	0.911413	pass	0.964295	pass	0.437274	pass
Universal	0.437274	pass	0.090936	pass	0.437274	pass	0.122325	pass
Approximate entropy	0.964295	pass	0.350485	pass	0.213309	pass	0.213309	pass
Random excursions	0.213309	pass	0.162606	pass	0.739918	pass	0.035174	pass
Random excursions variant	0.035174	pass	0.048716	pass	0.275709	pass	0.066882	pass
Serial	0.122325	pass	0.964295	pass	0.048716	pass	0.275709	pass
Linear complexity	0.162606	pass	0.834308	pass	0.017912	pass	0.911413	pass

**Table 2:** Performance comparison: proposed Direct QGT vs. traditional Dual-QFT method.

Metric	Component	Proposed QGT	Traditional Dual-QFT
MAE	Real (*q*_0_)	2.89 × 10^−15^	1.75 × 10^−15^
	Imaginary (*q*_1_)	2.11 × 10^−15^	1.98 × 10^−15^
	Imaginary (*q*_2_)	1.67 × 10^−15^	1.65 × 10^−15^
	Imaginary (*q*_3_)	1.99 × 10^−15^	1.75 × 10^−15^
Reconstruction SSIM	Real (*q*_0_)	1.0000	1.0000
	Imaginary (*q*_1_)	1.0000	1.0000
	Imaginary (*q*_2_)	1.0000	1.0000
	Imaginary (*q*_3_)	1.0000	1.0000
Execution Time (s)	Total	0.0431	0.0551

**Table 3:** SSIM values of RGB-D images decrypted with incorrect keys.

Cases	NYU0001		NYU0300		00666	
	RGB	Depth Map	RGB	Depth Map	RGB	Depth Map
*x*1 = *x*1 + 10^−15^	0.00053	0.01063	0.00093	0.01001	0.00044	0.00504
*y*1 = *y*1 + 10^−15^	0.00040	0.01059	0.00005	0.01003	0.00043	0.00479
*z*1 = *z*1 + 10^−15^	0.00011	0.01049	0.00143	0.01007	0.00004	0.00512
*w*1 = *w*1 + 10^−15^	0.00003	0.01065	0.00008	0.00978	0.00061	0.00481
*α*1 = *α*1 + 10^−15^	0.00005	0.01053	0.00081	0.00982	0.00018	0.00492
*β*1 = *β*1 + 10^−15^	0.00005	0.01061	0.00004	0.00981	0.00004	0.00457
*λ*1 = *λ*1 + 10^−15^	0.00068	0.01063	0.00056	0.01006	0.00029	0.00509
*γ*1 = *γ*1 + 10^−15^	0.00041	0.01059	0.00064	0.01006	0.00019	0.00488
*δ*1 = *δ*1 + 10^−15^	0.00029	0.01062	0.00053	0.00943	0.00013	0.00492
*τ*1 = *τ*1 + 10^−15^	0.00033	0.01072	0.00004	0.00945	0.00050	0.00504
*θ = θ* + 10^−3^	0.00232	0.01091	0.00501	0.01115	0.00250	0.00523
*x*2 = *x*2 + 10^−15^	0.00036	0.01061	0.00011	0.00976	0.00012	0.00513
*y*2 = *y*2 + 10^−15^	0.00005	0.01050	0.00040	0.00968	0.00054	0.00503
*z*2 = *z*2 + 10^−15^	0.00035	0.01051	0.00003	0.01013	0.00002	0.00537
*w*2 = *w*2 + 10^−15^	0.00048	0.01040	0.00185	0.00962	0.00005	0.00455
*α*2 = *α*2 + 10^−15^	0.00058	0.01052	0.00063	0.00958	0.00005	0.00498
*β*2 = *β*2 + 10^−15^	0.00013	0.01048	0.00005	0.00971	0.00053	0.00486
*λ*2 = *λ*2 + 10^−15^	0.00008	0.01061	0.00056	0.00981	0.00022	0.00507
*γ*2 = *γ*2 + 10^−15^	0.00017	0.01053	0.00084	0.00977	0.00008	0.00509
*δ*2 = *δ*2 + 10^−15^	0.00049	0.01065	0.00075	0.00972	0.00047	0.00481
*τ*2 = *τ*2 + 10^−15^	0.00023	0.01060	0.00052	0.00992	0.00042	0.00467

**Table 4:** Chi-square (*χ*^2^) test analysis.

Ciphertext	*χ* ^2^	*p*-Values	Decision (H = 0 or 1)
NYU0001	251.643	0.548	0; Accepted
NYU0300	279.326	0.141	0; Accepted
00666	266.476	0.298	0; Accepted

**Table 5:** Correlation statistics of plain RGB-D image and cipher image.

RGB-D Images			Horizontal	Vertical	Diagonal
NYU0001	Plaintext	Red channel	0.9928	0.9939	0.9970
		Green channel	0.9930	0.9946	0.9881
		Blue channel	0.9924	0.9946	0.9980
		Depth map	0.9785	0.9801	0.9781
	Ciphertext		0.0011	0.0028	−0.0007
NYU0300	Plaintext	Red channel	0.9787	0.9845	0.9640
		Green channel	0.9739	0.9832	0.9579
		Blue channel	0.9751	0.9824	0.9588
		Depth map	0.9699	0.9686	0.9559
	Ciphertext		−0.0008	0.0021	0.0036
00666	Plaintext	Red channel	0.9914	0.9949	0.9875
		Green channel	0.9918	0.9938	0.9867
		Blue channel	0.9855	0.9856	0.9723
		Depth map	0.9918	0.9943	0.9862
	Ciphertext		−0.0033	0.0070	0.0028

**Table 6:** The information entropies of the original RGB-D images and encrypted images.

Images	Plain RGB-D Image				Cipher Image
	Red Channel	Green Channel	Blue Channel	Depth Map	
NYU0001	7.5191	7.5710	7.6422	5.3855	7.9998
NYU0300	7.5727	7.7122	7.8412	6.7242	7.9998
00666	7.5148	7.4196	7.2877	4.9220	7.9999

**Table 7:** Values of NPCR and UACI for slight changes in color components or depth maps (%).

Cases	NYU0001		NYU0300		00666	
	RGB	Depth Map	RGB	Depth Map	RGB	Depth Map
NPCR	99.5998	99.6003	99.6042	99.6132	99.6038	99.6103
UACI	33.4717	33.4655	33.4778	33.4597	33.4632	33.4716

**Table 8:** Performance under the chosen-plaintext attack.

Image	Information Entropy	Correlation		
		Horizontal	Vertical	Diagonal
White	7.9994	−0.0022	0.0073	0.0060
Black	7.9994	0.0090	0.0016	−0.005

**Table 9:** The average execution times for encryption and decryption.

Dataset	File Size	Average Time for Encryption (s)	Average Time for Decryption (s)
SUNRGBD	427 × 561	0.2338	0.2396
Scenes	480 × 640	0.2709	0.2737

**Table 10:** Key space analysis.

Algorithm	Ours	[6]	[8]	[38]	[57]	[58]	[59]	[60]
Key Space	2^1007^	2^142^	2^628^	2^248^	2^299^	2^443^	2^472^	2^399^

**Table 11:** Performance comparison with existing image encryption methods.

Criteria		Ours	[6]	[38]	[58]	[61]	[62]
Information Entropy		7.9998	7.9993	7.9693	7.9985	7.9978	7.9981
Correlation	H	0.0017	0.0017	0.0024	0.0034	0.0073	0.0014
	V	0.0039	0.0009	0.0036	0.0037	0.0065	0.0031
	D	0.0023	0.0014	0.0029	0.0032	0.0082	0.0024
NPCR (%)		99.6053	99.6153	99.6121	99.6074	99.6092	99.6056
UACI (%)		33.4682	33.4750	33.4395	33.4665	33.4765	33.4525

**Table 12:** Comparison of time efficiency with other schemes.

Methods	Image Data (bit)	Encryption Time (s)	*ET* (Mbps)	*NCPB* (Cycles/Byte)
Ours	427 × 561 × 24 × 50 427 × 561 × 16 × 50 480 × 640 × 24 × 50 480 × 640 × 16 × 50	25.2381	43.3271	406
[57]	480 × 640 × 24 426 × 640 × 24 500 × 600 × 24 500 × 333 × 24 640 × 359 × 24	6.0900	5.0290	3977
[56]	512 × 512 × 8 512 × 512 × 24	6.5088	1.2888	15,518
[63]	256 × 256 × 24 512 × 512 × 24	6.1538	1.2780	16,275
[6]	512 × 512 × 8×6	0.9245	13.6105	1881
[58]	512 × 512 × 24 × 6	15.0089	2.5300	6956
[60]	512 × 512 × 8×4	0.6617	12.6773	2461

## Data Availability

The data used to support the findings of this study are available from the SUNRGBD Dataset (https://rgbd.cs.princeton.edu/, accessed on 17 July 2025) and the RGB-D Scenes Dataset (https://rgbd-dataset.cs.washington.edu/dataset/rgbd-scenes-v2/, accessed on 17 July 2025). The source code is not publicly available due to ongoing patent applications. The original contributions presented in this study are included in the article. Further inquiries can be directed to the corresponding authors.
